# Driving selective upcycling of mixed polyethylene waste with table salt

**DOI:** 10.1038/s41598-024-63482-1

**Published:** 2024-06-22

**Authors:** Mohamed Shaker, Syeda Shamila Hamdani, Tanyaradzwa S. Muzata, Muhammad Rabnawaz

**Affiliations:** School of Packaging, Michigan State University, 448 Wilson Road, East Lansing, MI 48824-1223 USA

**Keywords:** Chemical recycling, Upcycling, Wax production, Mixed polyethylene, Packaging, Chemistry, Materials science, Environmental impact

## Abstract

Advanced recycling offers a unique opportunity for the circular economy, especially for mixed and contaminated plastics that are difficult to recycle mechanically. However, advanced recycling has barriers such as poor selectivity, contaminant sensitivity, and the need for expensive catalysts. Reported herein is a simple yet scalable methodology for converting mixed polyethylene (high-density and low-density polyethylene recycled polyethylene) into upcycled waxes with up to 94% yield. This high yield was possible by performing the reaction at a mild temperature and was enabled by using inexpensive and reusable table salt. Without table salt, in otherwise identical conditions, the plastic remained essentially undegraded. These upcycled waxes were used as prototypes for applications such as water- and oil-resistant paper, as well as rheology modifiers for plastics. Their performance is similar to that of commercial wax as well as rheology modifiers. A preliminary economic analysis shows that the upcycled waxes obtained by this table salt-catalyzed approach offer three times more revenue than those reported in the literature. This pioneering discovery opens the door for a circular economy of plastics in general and polyolefins in particular.

## Introduction

Plastics have become an integral part of our daily lives, with applications ranging from packaging to lightweight manufacturing^1–4^. However, the reliance on packaging has also led to significant environmental issues. Less than 10% of all plastics that are ever produced are recycled^5^. The packaging industry alone accounts for 46% of all plastic waste^6^, and more than 50% of this packaging plastic waste is polyethylene (PE), such as high-density polyethylene (HDPE), low-density polyethylene (LDPE), and linear low-density polyethylene (LLDPE). Thus, boosting PE recycling will facilitate recycling efforts significantly and will mitigate the plastic waste issue.

For any recycling to be effective in the real world, it should meet three criteria: Firstly, there should be enough feedstock available in the municipal solid waste (MSW); secondly, there should be a way to clean and process the recycled material; and thirdly, there should be a valuable market for the recycled material. If any of these criteria are not met, recycling is complicated to get implemented in the real world. For example, rigid HDPE meets these criteria, and thus, HDPE bottles are recycled at a rate of 30%. Now, consider mixed PE, which is found in almost 50% by the total weight of all plastics in MSW (~ 50%)^7,8^, but has no market due to contamination and low performance of mixed PE.

PE pyrolysis to oil/gas is well-known to the scientific community. Interest has been focused on this area in recent years. For example, Huber et al. demonstrated hydroformylation of oil obtained via the pyrolysis of PE, leading to valuable aldehydes and alcohols derived from polyolefin waste^9^. Sadow et al. developed single-use polyethylene into high-value liquid products^10^. Meanwhile, Scott et al. explored the use of tandem catalysts for the upcycling of polyethylene, resulting in alkyl aromatics^11^ and α,ω-divinyl-functionalized oligomers^12^. Despite these advancements, considerable challenges remain, such as heterogeneity in plastic compositions and degradation during recycling^13^, high costs, the sensitivity of catalysts to contaminants, and achieving control over the products during chemical upcycling.

We chose to focus on upcycling waxes from mixed polyethylene because there are many uses for waxes, ranging from reality modifiers to lubricants, paper coating materials, agriculture surfactants, cosmetics, additives for road binders, and the manufacture of rubber. In other words, waxes are essentially a feedstock that can be used as a sole material, blended with other materials, or can be modified into a variety of different products^14,15^. For example, a commercial wax called Sasobit is often employed as a rheology modifier^16^. There is also a $5 billion market for wax in paper coating and cardboard applications^17^.

This study aims to convert mixed PE into upcycled waxes. There is great interest in wax formation as it can be used as a solo product or modified into something else. For example, an article in *AAA Science* has recently reported a wax recovery rate of up to 86% from polyethylene^18^. Although the importance of creating waxes from waste plastics is highlighted in this study, the complexity of the process and the reactor used may limit its commercial viability. Also, the study was performed on a small scale that did not allow for a large amount of wax formation, and it took 16 h to form waxes^18^.

Here, we present a cost-effective method for producing upcycled wax in 90% yield from recycled HDPE/LDPE mixtures. Recycled mixed PE is cheap (< 1 cent per lb) and difficult to recycle mechanically, and thus, it is an ideal feedstock for chemical recycling. We recently found a 92% wax yield in less than three hours from HDPE. However, HDPE is mechanically recyclable and has a good market value (~ 15 cents/lb)^19^. Also, the chemistry of linear and branched PE is different when it comes to pyrolysis. Branched LDPE is less thermally stable than linear HDPE. This lower thermal stability affects the yield of the waxes due to the rapid degradation of branched PE. This article reports our findings on the pyrolysis of mixed PE to waxes, applications for paper coating and rheology modifiers, and finally, provides a techno-economic analysis of this upcycling process.

## Experimental section

### Materials

HDPE and LDPE were supplied by Nova Chemicals (pellet form, the average size of each pellet is 0.12–0.13 inch), LLDPE (Dowlex 2056G, MFI—9 g/10 min) was purchased from Dow Chemicals, and polypropylene (PP, PD-1428) was supplied by Formosa Plastics Corp. The commercial Fischer–Tropsch wax Sasobit B52 was supplied by SASOL Chemicals, South Africa. The waste plastics were obtained from the Michigan State University (MSU) Surplus and Recycling Center, Michigan, United States. The waste plastics under study were separated to HDPE waste and LDPE wastes based on the number in the recycling symbol. The waste plastics were washed with detergent, then rinsed by tap water, and dried at room temperature for 24 h. The waste plastics were used after grinding to amorphous powder using a single speed mini cutting mill (20 MESH Screen).

### General procedure of wax recovery

An autoclave reactor was filled with 10.0 g of HDPE/LDPE mixture (virgin or waste) in different ratios (10–50% LDPE) and 1.0 g of NaCl (10 parts per hundred resins, referred to here and onward as 10 wt%). The autoclave reactor was completely wrapped with aluminum foil to maintain the pyrolysis temperature inside the reactor, and the reactor was placed in a pre-heating heating mantle at 425 °C (virgin HDPE/LDPE mixture) and 450 °C (waste HDPE/LDPE mixture) (see Table 1). The wax was recovered using three different approaches including: (1) dissolution in and evaporation of an organic solvent, such as chloroform; (2) cooling down and scraping out salt from the bottom of the solidified wax; and (3) hot-water dispersion followed by cooling, with the solid wax being collected from the top of the water surface (See SI, Figure S1).

**Table 1:** Pyrolysis conditions for HDPE/LDPE virgin mixture using (10 wt%) NaCl at 450 ± 5 °C, pyrolytic waxes yield, and characterization data.

Pyrolysis Condition			Pyrolytic Crude solid wt%^*a*^			Wax thermal properties ^*c*^			Wax Molecular weight	
**Sample**	HDPE/LDPE ratio	Time	Gas wt%	Volatile materials^*b*^	Wax^*b*^	*T*_m_ (°C)	*T*_c_ (°C)	*T*_*d*_ (°C)^*b*^	Mw^c^ g/mol	Mw^d^ g/mol
**Sasolwax B52**	Commercial wax was used as a reference					97	90	313		
**V/50% powder**	50/50 **Before pyrolysis**	Powder mixture				131	115	435		
**V/50% blank**	50/50 **without salt**	1.00 h*	No significant degradation (%)			127	111	437		
V/10%	90/10	3.00 h	4	96		109	96	420	608	1011
				6	90					
**V/20%**	**80/20**	3.00 h	5	95		101	88	426	544	852
				17	78					
V/30%	70/30	2.00 h*	4	96		105	90	415	544	975
				13	83					
V/40%	60/40	1.00 h*	3	97		117	102	433	567	1429
				6	91					
V/50%	50/50	1.00 h*	4	96		118	103	438	528	1441
				5	91					
V3	70/30	3.00 h	15	15	70	Viscous wax applied in paper coating				
V4	60/40	2.00 h*	25	15	60					
V5	50/50	2.00 h*	35	20	45					

V: Virgin HDPE/LDPE mixture.

V/number%: the percentage denotes LDPE by wt. in HDPE blend.

^a^Isolated yield after scraping the salt layer.

^b^Calculated from TGA analysis.

^c^Molecular weights of the longest hydrocarbon chain determined by GCMS analysis.

^d^Predicted based on the melting temperature using: https://www.engineeringtoolbox.com/hydrocarbon-boiling-melting-flash-autoignition-point-density-gravity-molweight-d_1966.html.

*Denotes samples that were treated with a shorter pyrolysis time.

### Use of upcycled waxes in laboratory-controlled polyolefins (L-PO) as rheology modifiers

Laboratory-controlled polyolefins (L-PO) comprised three types of PE (HDPE, LDPE, and LLDPE) and PP. The PE/PP composition was chosen to be 62.5/37.5 wt/wt to mimic the PE and PP present in U.S. municipal solid waste (MSW)^20^. L-PO pellets were prepared from the PE above/PP blends by a single screw extruder (SSE) at a temperature profile of 190, 180, 170, and 160 °C utilizing a screw speed of 20 rpm. The bulk polymer strands were then pelletized into small pellets. These pellets were then mixed with different concentrations of the wax and subsequently processed via a laboratory-sized DSM micro-compounder at a temperature of 180 °C for 10 min to ensure adequate mixing of the polyolefins and the waxes. The L-PO/wax polymer strands were cut into small pellets for melt flow evaluation.

### MFI measurements

The melt flow index (MFI) of the L-PO/wax was determined by inserting the pellets into a Ray Ran melt flow indexer at a temperature of 230 °C using a weight of 2.16 kg. The MFI value was taken from an average of five samples. Minitab software was used for statistical analysis of the MFI data.

### Paper coating with upcycled waxes

Kraft paper was coated with a 5% starch solution and air dried for 24 h to obtain a paper substrate for applying upcycled waxes. The dried starch-coated paper was trimmed to dimensions of 20 × 14 cm^2^ and fixed on the aluminum plate using a piece of tape to prepare paper substrates. The coating solution was prepared by dissolving 1.1 g of wax material in chloroform using a 25 mL vial, which was heated and stirred at 60 ℃ for 2–3 min to obtain a clear solution. The resultant clear solution was cast onto paper substrate using a silicon spatula and subsequently air-dried in a fume hood for 10 min before drying in an oven at 65 ℃ for 20 min. After removal from the range, paper samples were airdried at room temperature for 24 h before water and oil resistance analysis.

## Results and discussion

**Figure 1:**
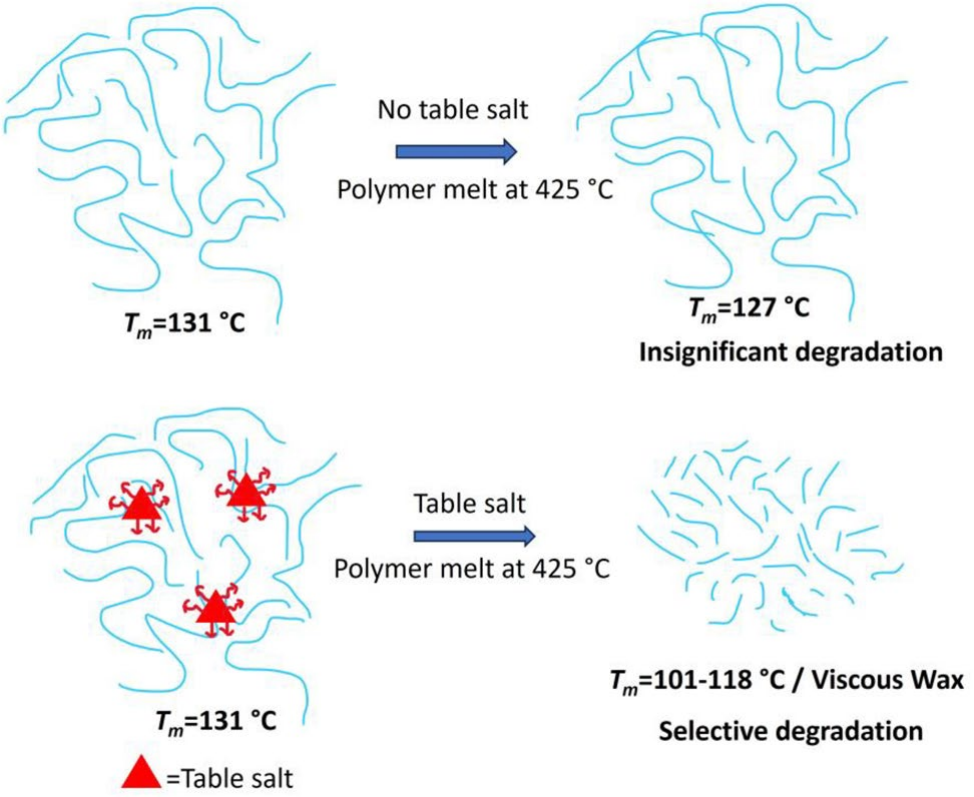
Schematic illustration of mechanism suggesting the role of table salt in the chemical upcycling of waste plastics by enabling mild-temperature pyrolysis.

This study is aimed at a comprehensive and significant problem, namely the advanced recycling of mixed contaminated PE. Branched PE (LDPE) is thermally less stable than linear PE (HDPE), and the conditions required to obtain wax from mixed PE are likely to be different than those needed for HDPE alone. We initiated this study by performing thermogravimetric analysis (TGA) of HDPE and LDPE blends. To understand the effect of table salt, we conducted a comparative TGA analysis of HDPE/LDPE blends both in the presence and absence of table salt (a good thermal conductor with a high heat capacity) and silica (a low thermal conductor with a low heat capacity). We hypothesize that table salt reduces the degradation temperature thanks to its high heat capacity and good thermal conductivity. For example, the thermal conductivity of PE is nearly 0.4 W/(m K)^21^, while that of sodium chloride is almost 3.5 W/(m K)^22^. Therefore, sodium chloride facilitates the transfer of heat more readily. In addition, the high heat capacity of sodium chloride allows it to absorb more heat that is then transferred to the plastic. These high heat spots trigger degradation while allowing a low overall plastic melt temperature to be maintained, and this leads to milder conditions for pyrolysis as shown in Fig. 1. As a result, pyrolysis can be performed at lower temperatures with more control over product selectivity. In contrast, we saw no real decrease in the *T*_d_ when silicon dioxide was added to the HDPE/LDPE blends. See Supporting Information (SI), Figure S2.

### Virgin HDPE/LDPE mixture chemical upcycling into wax

Virgin HDPE/LDPE mixtures where the LDPE content was varied from 10, 20, 30, 40, and 50 by wt% were used as feedstocks to produce upcycled waxes. Unless specified otherwise, the pyrolysis was performed in the presence of 10 wt% of table salt. The detailed composition and the physical properties of the obtained upcycled waxes are listed in Table 1. Waxes were recovered using various techniques, including hot-water dispersion, solvent dissolution-evaporation, or scratching salt off the bottom of solidified wax. The latter of these three methods was particularly efficient because the salt and wax could be simply separated, and both recovered wax and salt were ready for further use.

**Figure 2:**
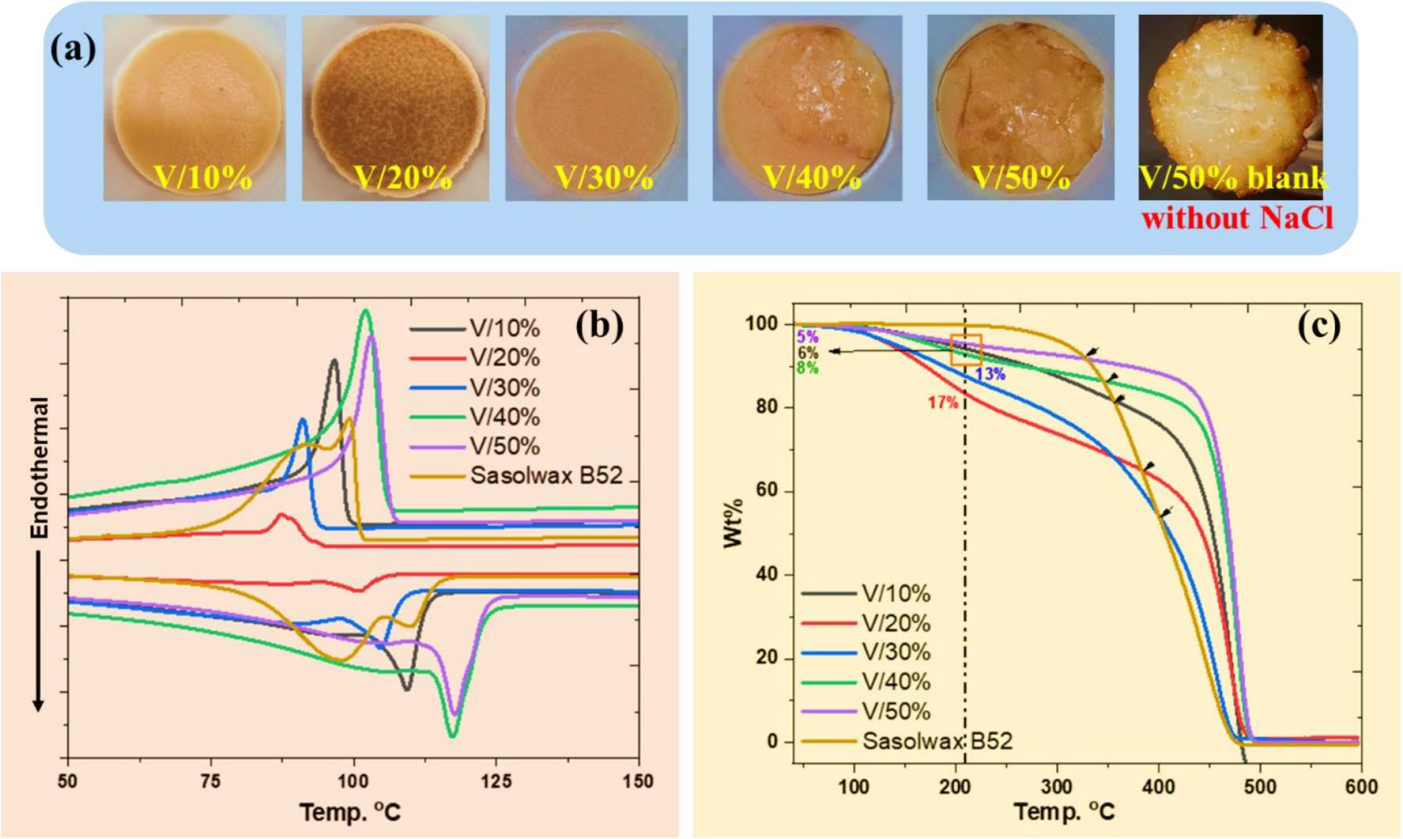
Upcycled waxes from virgin HDPE/LDPE mixture. (**a**) Photographs of the pyrolytic waxes obtained from virgin HDPE/LDPE mixtures; V (10%, 20%, 30%, 40% and 50%); and V/50% blank (without salt) 1 h/pyrolysis. (**b**) DSC curves of the waxes obtained from the HDPE/LDPE mixtures compared with Sasolwax B52. (**c**) TGA curves of the obtained waxes from virgin HDPE/LDPE mixtures compared with Sasolwax B52, with a heating rate of 10 °C/min under nitrogen atmosphere.

As shown in Table 1, high yields of waxes were recovered (≥ 90%). To obtain viscous waxes (V3-V5), virgin HDPE/LDPE mixtures were heated with 10 wt% table salt for three h in the case of the 70/30 mixture (Table 1, labeled as V3) and for two h for the 60/40 and 50/50 HDPE/LDPE mixtures (Table 1, labeled as V4 and V5). Photographs of the obtained pyrolytic viscous waxes are shown in Figure S4, SI. The virgin HDPE/LDPE (50:50) mixture did not produce wax products under the blank pyrolysis methodology (without adding NaCl). Figure 2a shows the photograph of the solid obtained from the blank pyrolysis (V/50% blank). The thermal characteristics of the recovered product (V/50% blank) show that it is not wax but rather, a moderately degraded HDPE. ^1^H-NMR analysis of the soft waxes reveals that they are primarily saturated hydrocarbon chains contaminated with about 1–2% alkene content assigned by the presence of unsaturated protons at 4.9, 5.4, and 5.8 ppm and there are no aromatic protons found. The viscous waxes contaminated with 1 mol% of aromatic content assigned at 7.0–7.3 ppm (see Figures S8-S14).

The thermal properties of the recovered waxes were determined using both differential scanning calorimetry (DSC) and TGA analysis (Fig. 2b,c), and the data are summarized in Table 1. In general, all obtained waxes possessed melting points (*T*_m_) over 100 °C, with a temperature range of 101–118 °C. The DSC analysis showed that the thermal properties of the V/10%, V/20%, and V/30% recovered waxes match those of the commercial Sasolwax B52 (Fig. 2b), considering that the V/20% and V/30% waxes are contaminated with a high wt% of volatile materials, 17 and 13%, respectively (Fig. 2c). Heating the HDPE/LDPE mixture (50:50) at the optimized temperature for just 1 h yielded the recovered V/50% wax which showed the highest* T*_m_ and cold crystallization temperature (*T*_c_) values at 118 and 103 °C, respectively. It is worth mentioning that the V/40% wax almost has the same *T*_m_ and *T*_c_ of its V/50% counterpart, thus confirming that the simple change in the mixture ratio to (60:40), does not affect the thermal properties of the resulting wax under the same pyrolysis conditions. The decrease in the *T*_m_ and *T*_c_ of the recovered solids clearly indicated the successful formation of waxes from virgin HDPE/LDPE mixtures.

From the TGA analysis, it is evident that the obtained waxes are more thermally stable compared with the commercial Sasolwax B52 (Fig. 2c). All recovered waxes possessed thermal degradation temperature (*T*_d_) over 415 °C while the referenced Sasolwax B52 decomposed at 313 °C (Table 1). The most thermally stable waxes are V/40% and V/50%, which decomposed at 433 °C and 438 °C, respectively, considering the short pyrolysis time (1 h). As reported, the pure paraffin wax has a one‐step thermal decomposition profile which occurred at 208 (± 2)°C^23–25^. Consequently, 210 °C was chosen as a reference point to determine the actual recovered wax yield and the ratio of contaminated volatile materials, so the losses observed below these temperatures were considered as liquid volatile products and the losses observed above this temperature of 210 °C were taken as solid wax. As mentioned above, at 210 °C, the V/20% and V/30% waxes were contaminated with high wt% of volatile materials, 17 and 13%, respectively. Meanwhile, the amounts of volatiles in the V/10%, V40%, and V50% waxes are 6%, 6%, and 5%, respectively (see Table 1, Fig. 2c). It is worth mentioning that, at 400 °C the V/30% wax has identical thermal stability to that of Sasolwax B52 by losing 46% of its analyzed weight, while other waxes such as V40% and V50% still showed high thermal stability and underwent modest weight losses of 18% and 12%, respectively (Fig. 2c). Gas chromatography-mass spectrometry (GC–MS) analyses were performed but did not provide any useful information for the hard waxes (V/10%-V/50%) that had low solubility and vapor pressure see Figures S23–S27. However, soft waxes (V3-5) displayed a consistent series of saturated hydrocarbon peaks from C15 to C35, as shown in Figures S28–S30. It is evident from our NMR and GC–MS analysis that the upcycled waxes are primarily saturated alkanes.

### Upcycled wax from waste HDPE/LDPE mixed plastics

**Figure 3:**
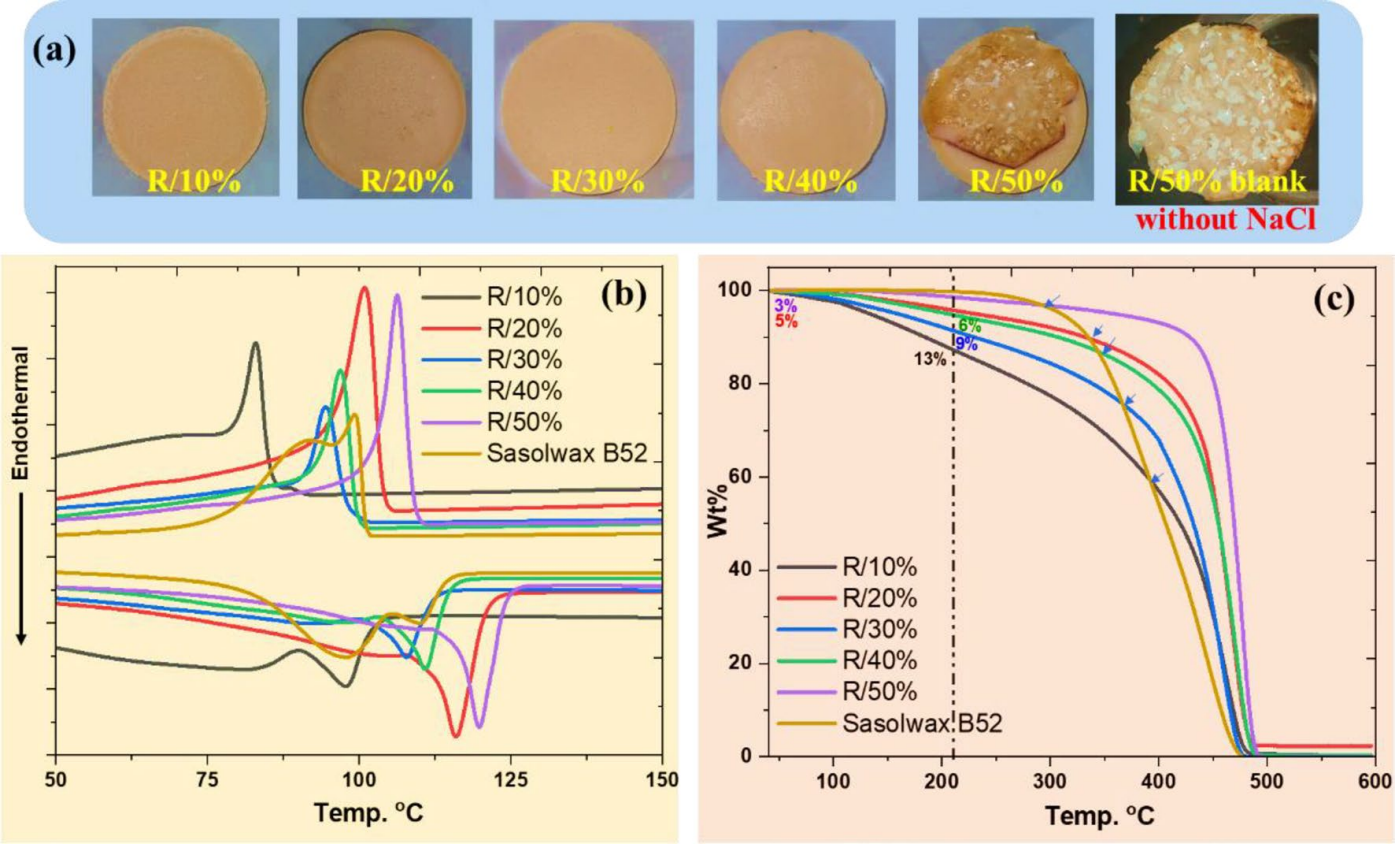
Upcycled waxes from waste HDPE/LDPE mixtures. (**a**) Photographs of the pyrolytic wax obtained from the waste plastics mixtures; R (10%, 20%, 30%, 40% and 50%); and R/50% blank (without salt), 1 h/pyrolysis. (**b**) DSC curves of the pyrolytic wax obtained from the waste plastics HDPE/LDPE mixtures compared with those of Sasolwax B52. (**c**) TGA curves of the pyrolytic wax obtained from the waste plastics HDPE/LDPE mixtures compared with those of Sasolwax B52. The DSC and TGA experiments were performed at a heating rate of 10 °C/min under nitrogen atmosphere.

The pyrolysis of waste HDPE/LDPE mixture (labeled **R**) with different mixing ratios (from 10 to 50% of waste LDPE) was performed in the presence of 10 wt% salts in an autoclave at 450 °C for the optimized pyrolysis operating time as shown in Table 2. The upcycled waxes (with a high *T*_m_) R/10%, R/20%, R/30%, R/40%, and R/50%, which had been obtained from waste mixtures, were recovered after heating for periods in the range of 1.00–2.00 h, and the resultant yields of the crude waxes were ≥ 94% (Table 2)*.* The wax yield was lower with a longer pyrolysis time because of the further degradation of the wax into oil. To obtain viscous waxes, the pyrolysis time was increased to 4 h which was longer than the reaction time employed for virgin mixtures, but it is presumably due to the high molecular weights of recycled LDPE/HDPE. The yields of the recovered viscous waxes were 79%, 72%, and 50% for R3, R4 and R5, corresponding to 70/30, 60/40, and 50/50 waste HDPE/LDPE mixtures, respectively (Table 2). Photographs of the obtained pyrolytic viscous waxes R3, R4, and R5 are shown in Figure S5, SI. Under the blank pyrolysis methodology (in the absence of NaCl), the waste HDPE/LDPE (50:50) mixture did not create wax product; Fig. 3a shows the photograph of the solid obtained from the blank pyrolysis (R/50% bank) which has a non-uniform appearance with some remaining waste on the top. Figure S7 in the SI shows the thermal characterization data for the recovered product (R/50% blank). These findings confirm our recent investigation and demonstrate the critical impact that NaCl plays in enhancing polyolefin pyrolysis^26^.

**Table 2:** Pyrolysis conditions for waste HDPE/LDPE mixtures using (10 wt%) NaCl at 450 ± 5 °C, pyrolytic waxes yields, and characterization data.

Pyrolysis Condition			Gas wt%	Pyrolytic Crude solid wt%^*a*^		Wax thermal properties ^*c*^			Wax Molecular weight	
Sample	HDPE/LDPE waste ratio	Time (h)		Volatile materials ^*b*^	Wax^*b*^	*T*_m_ (°C)	*T*_c_ (°C)	*T*_*d*_*(*°C)^*b*^	Mw ^c^ g/mol	Mw ^d^ g/mol
**Sasolwax B52**	Commercial wax used as a reference					97	90	313		
**R/50% powder**	50/50 **no pyrolysis**	Powder mixture				137	117	426		
**R/50% blank**	50/50** without salt**	1.00*	≥ 99% solid product (%)			125	110	438		
R/10%	90/10	2.00	5	95		97	83	333	592	818
				13	82					
R/20%	80/20	1.50	2	98		116	101	411	544	1417
				5	93					
R/30%	70/30	1.50	2	98		107	94	389	528	993
				9	89					
R/40%	60/40	1.25	2	98		111	97	412	576	1114
				6	92					
R/50%	50/50	1.00*	3	97		119	106	433	512	1453
				3	94					
R3	70/30	4.00	10	11	79	Viscous wax applied in paper coating				
R4	60/40		17	11	72					
R5	50/50		35	15	50					

R: Recycled/waste HDPE/LDPE.

R/number%: the percentage denotes LDPE by wt in HDPE blend.

^a^Isolated yield after scraping the salt layer.

^b^Calculated from TGA analysis.

^c^Molecular weights of the longest hydrocarbon chain determined by GCMS analysis.

^d^Predicted based on the melting temperature using: https://www.engineeringtoolbox.com/hydrocarbon-boiling-melting-flash-autoignition-point-density-gravity-molweight-d_1966.html.

*Denotes samples that were exposed a smaller pyrolysis time.

The ^1^H-NMR analysis confirmed that the upcycled wax from waste HDPE/LDPE mixed plastics are almost identical with the waxes recovered from the virgin HDPE/LDPE mixtures, see SI, Figure S15–S21. For example, the ^1^H-NMR charts of R/50% and V/50% as soft waxes as well as R5 and V5 as viscous waxes are completely identical, see SI, Figure S22. As indicated earlier, GC–MS was only effective for the viscous waxes (R3, R4 and R5), which exhibited light hydrocarbons up to C35 (based on standard alkane calibration).

The *T*_m_ and *T*_c_ of the resulting waxes were determined by DSC analysis (Fig. 3b). As mentioned earlier, 1.00–2.00 h of heating at 450 °C was enough to depolymerize waste HDPE/LDPE mixtures (R) and R/10% resulted waxes showed the lowest *T*_m_ and *T*_c_ values of 97 and 83 °C, respectively (Table 2). Meanwhile, the highest* T*_m_ and *T*_c_ are (116, 101 °C) and (119, 106 °C) corresponding to R/20% and R/50%, respectively, considering that the pyrolysis of R/20% was run 30 min more than R/50% mixture. The thermal properties of the R/30% and R/40% recovered waxes are similar to those of the Sasolwax B52 commercial wax. The *T*_m_ of R/30% and R/40% recovered waxes are 107 and 111 °C, respectively, and is 110 °C for Sasolwax B52. Similarly, the *T*_c_ of Sasolwax B52 is in range of 92–99 °C and those for the R/30% and R/40% recovered waxes are 94 and 97 °C, respectively (Table 2).

TGA analysis was used to determine the thermal stability and whether any volatile oils were present in the recovered crude waxes (Fig. 3c). Supposing that wax decomposition started after 210 °C^22–24^, it appears that the R/10%, R/20%, R/30%, R/40%, and R/50% recovered waxes lost 13%, 9%, 6%, 5%, and 3%, respectively, of their initial weights. These losses can be attributable to volatile contaminant materials. As a result, Table 1 shows that the wax contents for R/10%, R/20%, R/30%, R/40%, and R/50%, respectively, are 82, 93, 89, 92, and 94 wt% after the volatile components are subtracted. The R/10% wax was less thermally stable compared with the other recovered waxes and Sasolwax B52 as well, while the waxes recovered from R/50% showed significant thermal stability considering the short duration of the pyrolysis process. The recovered waxes from R/20%, R40%, and R/50% possessed degradation temperatures (*T*_d_) over 400 °C which is significantly higher than the commercial Sasolwax B52 (which is 313 C°), thus offering the recovered waxes an advantage for applications such as rheology modifiers at high-temperature processing methodologies. The waxes recovered from R/30% and R/40% waxes have the same degradation temperature as that of Sasolwax B52 at 367 and 344 °C, respectively.

### Paper coating application

**Figure 4:**
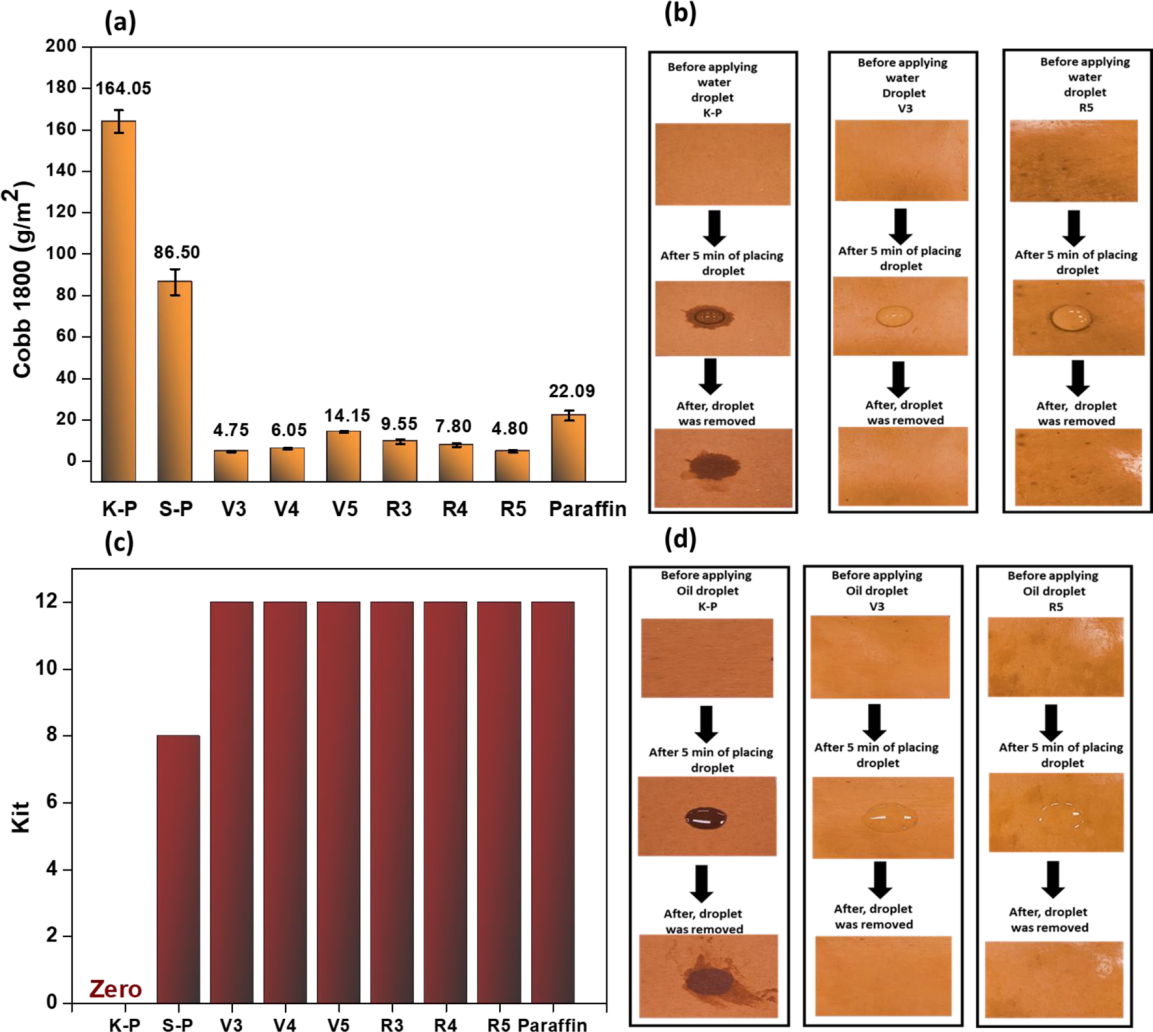
Upcycled waxes are used as a coating material for paper products. (**a**) Cobb1800 values (g/m^2^) of coated and control samples. Uncoated kraft paper sample (K-P), paper coated with Vn (wax recovered from virgin plastic, n = 3, 4, & 5), paper coated with Rn (wax recovered from recycled plastic, n = 3, 4, & 5) and paper coated with paraffin wax used as a control. (**b**)Water droplet behavior on paper samples capturing images before the application of the droplet, 5 min after the application of the droplet, and after removal of droplet after 5 min. (**c**) Kit ratings for uncoated and coated paper samples. (**d**) Oil resistance visual representation expressed by castor oil droplet behavior on paper samples including uncoated kraft paper sample (K-P), paper coated with V3 (wax recovered from virgin plastic), and paper coated with R5 (wax recovered from recycled plastic). The behavior of a castor oil droplet was recorded by capturing images before the application of droplet, 5 min after the of application of the droplet, and after the removal of the droplet after 5 min.

The coated papers were screened for their water resistance via Cobb1800 tests, and the results are shown in Fig. 4a, b. The paper samples, uncoated kraft paper (K-P), and 5% starch-coated paper and paraffin wax-coated paper (Paraffin) were used as controls. A very high Cobb1800 value (i.e., 164.05 ± 5.44 g/m^2^) was observed for the uncoated kraft paper due to the highly porous and hydrophilic nature of cellulose fibers^27^. Although a starch layer covered this porous surface, The starch-coated paper still possessed a slightly higher Cobb1800 value despite bearing a starch layer that covered its porous surface due to the hydrophilic nature of starch^28^. The application of paraffin wax has reduced the water absorption, thus enhancing the water resistance up to a Cobb1800 value of 22.09 ± 2.41 g/m^2^ due to hydrophobic nature of paraffin wax. Water resistance was increased by applying the selected upcycled waxes (depending on their solubility in chloroform solvent) from the virgin and recycled series of samples. Among the virgin series, the best-performing sample was V3, which showed the lowest Cobb1800 value of 4.75 ± 0.35 g/m^2^ and an almost equal value of 4.80 ± 0.56 g/m^2^ was exhibited by the R5 sample (the best performer) from the recycled-series. The highest Cobb1800 value among the wax-coated paper samples was 14.15 ± 0.35 g/m^2^ shown by sample V5 among tested samples which was still much lower than those of many commercial benchmarks^29^, and paraffin-coated paper, suggesting its applicability as a paper coating. These results show that waxes obtained from recycled plastics have water resistance almost like waxes obtained from virgin polymers, thus suggesting that waxes obtained from waste recycled plastics do not lose their integrity.

The water resistance offered by coated paper samples was visualized by water droplet tests (Fig. 4b); the samples with the best Cobb1800 values were selected for this test (V3and R5). During each test a droplet of deionized water was placed on the surface of coated paper sample and images were recorded after five minutes and then the droplet was wiped off. Images were recorded again and compared with those taken before the application of the droplet. The photos of coated paper samples were evaluated based on compassion with uncoated kraft paper to find changes in water absorption. For uncoated paper (K-P) a dark stain began to appear quickly after the placing of the droplet. This stain was still visible after the droplet had been wiped away, indicating that the uncoated paper had poor water resistance. In contrast, the coated paper was able to support the droplet for 5 min (without it becoming absorbed into the paper) and there was no stain visible following the removal of these droplets (shown by sample V3 and R5), thus demonstrating that the water resistance was improved after application of the coating.

The oil resistance of coated paper samples was measured by a standard kit test following a previously reported procedure (Fig. 4c,d)^30^. Uncoated paper exhibited a kit rating of zero because it failed the test performed with kit solution 1, which left a stain behind. The kit value was improved with the application of starch layer on kraft paper (S-P) up to 8. There was further improvement in kit value with the wax-coated samples. All the wax-coated samples showed kit value of 12, which is equal to that exhibited by the paraffin-coated paper (used as a control), thus indicating these paper samples had highly oil resistant surfaces.

Like water resistance, oil resistance was visualized by recording castor oil droplet behavior on the surfaces of various coated paper samples. The same paper samples were chosen for testing which had been subjected to the water droplet tests (Fig. 4d). The oil droplet left a dark stain on uncoated paper. In contrast, no stains were visible on the wax-coated samples after an oil droplet had been placed on its surface and subsequently wiped away, thus suggesting that they have strong potential as oil repellent packaging products.

### Application of wax as rheology modifiers

One of the challenges encountered in efficiently recycling mixed plastics is the issue of inconsistent MFI values. Usually, waste plastics comprise different types of polymers having varying MFI values. Plastics that are processed via injection molding usually require high MFI values and the opposite is the case for plastics that are processed via extrusion. Rheological modifiers can be used to improve the processibility of waste plastics^31^.

**Figure 5:**
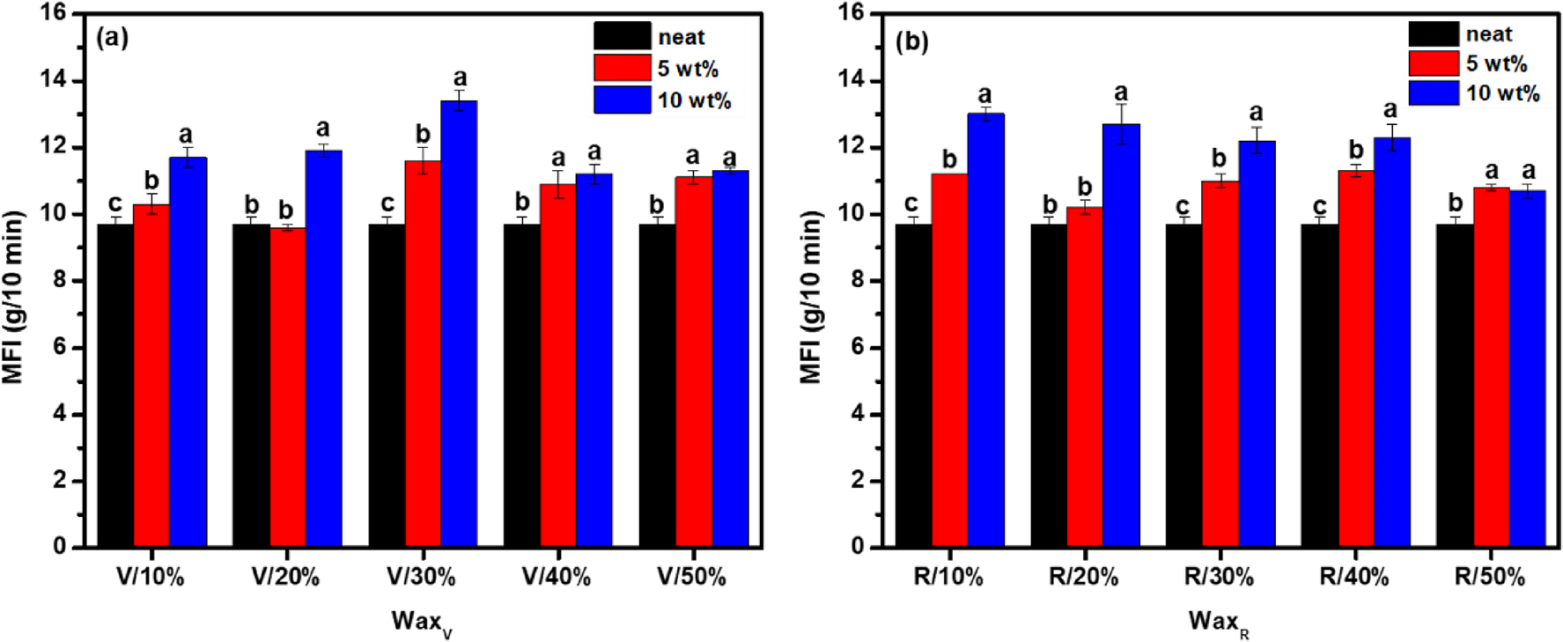
The MFI values of the laboratory-controlled polyolefins in the presence of (**a**) wax derived from virgin polymers (**b**) wax derived from waste recycled polymers. *Means that do not share the same letter are significantly different.

The effect of the wax derived from a stream of waste plastics in its ability to alter the rheological properties of laboratory-controlled polyolefins was investigated. An increase in the MFI values when the concentration of the waxes was increased was observed for all laboratory-controlled polyolefins (L-POs) samples (see Fig. 5). Virgin (**V**) waxes impact on the MFI of (L-POs) are shown in Fig. 5a. Recycled (**R**) waxes impact on the MFI of (L-POs) are shown in Fig. 5b. This increase in the MFI of L-POs in the presence of the waxes is due to the reduction in viscosity. It is well known that MFI is inversely proportional to viscosity, the wax acts as a plasticizer in disrupting the entanglements in the polyolefin polymer structure leading to a free flow of the polymer chains^32^. The practical significance of MFI modifiers is that such flow modifiers are widely used for both virgin and recycled plastic processing, and the use of upcycled waxes for such purpose creates another market for upcycled plastics.

### Economic analysis

**Figure 6:**
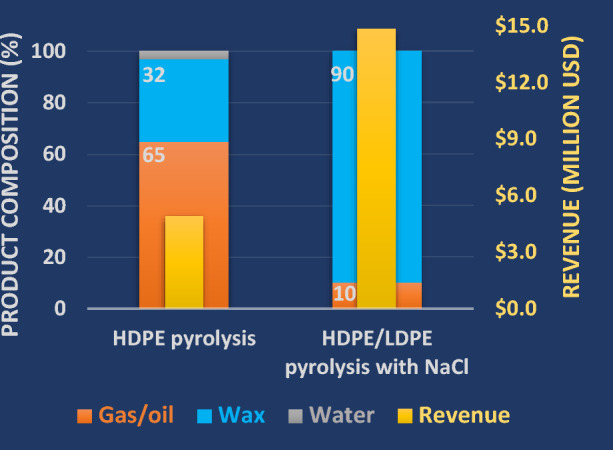
Economic analysis comparing the anticipated revenue for our methodologies (90% wax recovery) versus those of a literature benchmark^14^. *Note*: This TEA is preliminary for only a 10 g scale and significant deviations may occur when the reaction is scaled up. The price of natural gas is taken as 2.64 $/MMBtu and gasoline as 3.65 USD/gal.

Figure 6 shows our preliminary economic analysis for the upcycled wax products. Detailed analyses are provided in Tables S1 and S3 in the SI. We set the following parameters for this analysis: A reactor was chosen with an annual capacity of 8400 tons of waste plastic, with 10 wt% NaCl. Given that the mass yields of wax and pyrolysis vapors are 90% and 10%, respectively, an annual production of 7,560 tons of wax can be achieved. Prices for the upcycled waxes for rheology modifiers and wax-coated paper were set at $2 per kilogram on a one-ton scale. The price for recycled mixed plastic waste was set at $1.01 per pound. With 90% of wax recovery, our system would generate $14.82 million in revenue a year, which is three times more than the revenue generated from the HDPE control pyrolysis with a wax yield of 32%^14^. This initial analysis confirms that value-added upcycled waxes have economic benefits compared to gas/oil products and also promotes a true plastic circular economy.

In the future, we will investigate to see if any corrosion is caused by NaCl. NaCl can only facilitate corrosion if moisture or water is present in the system or if sodium chloride dissociates into Na and Cl ions. However, at this high pyrolysis temperature there is no moisture present, and thus NaCl remains intact. In addition, NaCl is very stable at temperatures up to 600 °C and does not form any corrosive HCl. We should not confuse NaCl with polyvinyl chloride (PVC). The difference is that PVC decomposes and creates HCl at 250–300 °C, so NaCl is clearly different and stable^32^. Both moisture and NaCl degradations are absent in the NaCl-assisted PE pyrolysis because PE is a hydrocarbon which lacks oxygen content, and also due to the absence of water in the reactor. In addition, any initial moisture can easily be removed from the reactor by opening the valves during heating from room temperature to 200 °C where no degradation occurs. Moreover, the pyrolysis employed in our case is performed well below the degradation temperature of NaCl. During pyrolysis, the table salt did not melt and did not produce Cl radicals or Cl^−^ ions in the absence of moisture. The lack of any Cl radical or Cl^-^ ion formation was supported by GC–MS analysis, as no chlorinated hydrocarbons were detected when EI ionization was used. The absence of chlorohydrocarbons is confirmed by the lack of observed M and M + 2 peaks at a 3:1 ratio, as would be expected for Cl35 and Cl37 (SI, Figure S39, S40). This supports the inertness of the Cl. For all these reasons, we do not expect corrosion or reactions between chorine and hydrocarbons to occur, but we will explore this in the future. In addition, if corrosion were to be evident in the salt-assistant pyrolysis approach, various strategies, such as applying ceramic and inorganic coatings or utilizing carbon-coated reactors, could be implemented to counteract potential corrosion issues^34–36^. For example, a practical approach addressing corrosion concerns involves employing a TiO_2_ lining within steel reactors for the mass production of terephthalic acid, where millions of tons are produced annually^37^. The combination of bromine and acetic acid used in the synthesis process is highly corrosive, thus necessitating the adoption of reactors that are specifically lined with titanium dioxide to prevent corrosion. Future exploration of the corrosion and the mechanism to understand the exact roles and synergies presented by sodium chloride in the pyrolysis process are planned, which be published in the near future.

## Conclusions

In summary, we have demonstrated an efficient method to produce upcycled waxes with quantitative yield from both virgin and waste HDPE/LDPE mixtures. This quantitative yield was possible by reducing the pyrolysis temperature. TGA analysis confirmed that table salt lowered the temperature for the degradation of mixed polyolefins. The recovered waxes were used to fabricate water- and oil-resistant papers. The results show that our waxes obtained from recycled plastics have water resistance similar to those of waxes obtained from virgin plastics, with excellent water repellency (Cobb1800 value of 4.80 ± 0.56 g/m^2^) and oil resistance (12/12 kit rating). In contrast, the uncoated paper had a Cobb1800 value of 164.05 ± 5.44 g/m^2^ and a kit rating of 0. The wax prepared from HDPE/LDPE mixed waste was able to significantly increase the melt flow index of the L-POs at high concentrations (5 and 10 wt%), thus confirming that recycled waxes made from waste plastic can be utilized to modify the melt flow characteristics of mixed polyolefins. Economic analysis shows that using the table salt approach can create up to $15 million in revenue from a reactor doing the pyrolysis of 8,400 tons of plastic per year, which is three times more than that performing the same pyrolysis without using table salt. These discoveries will strengthen the plastics industry's circular economy and safeguard the environment from plastic waste.

### Supplementary Information

Supplementary Information.

## Data Availability

Data is provided within the supplementary information file.
