# Supplementary material for: Driving selective upcycling of mixed polyethylene waste with table salt
Source: Sci Rep. 2024 Jun 22;14:14371. doi: 10.1038/s41598-024-63482-1 (PMC11193746; doi:10.1038/s41598-024-63482-1)
Supplement: Supplementary file 1 — Supplementary Information. [file 41598_2024_63482_MOESM1_ESM.docx]

**Supplementary Information**

**For**

**Driving Selective Upcycling of Mixed Polyethylene Waste with Table Salt**

Mohamed Shaker, Syeda Shamila Hamdani, Tanyaradzwa S. Muzata, and Muhammad Rabnawaz*

■ **AUTHOR INFORMATION**

School of Packaging, Michigan State University, 448 Wilson Road, East Lansing, MI 48824-1223, USA

***Corresponding author:**

Muhammad Rabnawaz

School of Packaging, Michigan State University

East Lansing, Michigan 48824-1223, United States

orcid.org/0000-0002-4576-1810

Email: rabnawaz@msu.edu;

Tel: +1-517-432-4870

**Paper Coating Procedure**

Wax (1.1g) was dissolved in 2.2 mL of chloroform by heating at 60 °C until a clear solution was obtained. Paper that had previously been coated with starch (5%) was trimmed to dimensions of 20 × 15 cm^2^ and wax solution was applied onto its surface. The coated paper was subsequently dried in an oven at 65 ℃ for 30 min. The samples were cooled down after they had been removed from the oven and kept at room temperature for 24 h prior to other testing.

#### **Liquid water resistance**

The standard TAPPI protocol, T441, was used to record the water resistance of paper samples against liquid deionized water via Cobb Test performed for 1800 s. The weighed paper sample was placed in a Cobb sizing tester (Büchel BV Inc. Utrecht, Netherlands) and 100 mL of deionized water was added to it. The sample was weighed again after the test and the difference in weight was related to the Cobb1800 value, and the results were expressed in units of grams per square meter (g/m^2^).

Also, we performed water droplet test by placing a 0.1 mL droplet of water on the surface of each paper sample and the results were recorded by capturing images after the interval of 5 min and after the droplet was wiped off the paper.

A Cobb sizing tester (Büchel BV Inc. Utrecht, Netherlands) was used to record water resistance on coated paper samples in comparison to uncoated kraft paper. The TAPPI standard method T441 has been used for this analysis and the Cobb1800 value was recorded where a paper sample was exposed to 100 mL of deionized water for a time of 1800 seconds. The paper sample was weighed before pouring water on to it and after the test. The difference between the weight of the paper before and after analysis was recorded as the Cobb1800 value and the result was expressed in units of grams per square meter (g/m^2^).

The water droplet test was carried out by placing a droplet with a volume of 0.1 mL on paper surface and recording images before placing, 5 minutes after it had been placed on the paper, and after the droplet had been wiped off from the paper surface.

**Oil Resistance**

The oil resistance of the developed coated papers was established by recording their kit ratings following a standard TAPPI T559 method. A series of kit solutions with kit ratings of 1-12 was prepared using different ratios of *n*-heptane, toluene, and castor oil. A 0.1 mL droplet of the test solution was then placed on a piece of paper sample for 15 s to check for the appearance of any dark spots, which would indicate failure of the paper to resist that kit solution. If there no spot had appeared for a test solution, the paper was considered to have passed the kit rating test for that liquid. The highest number of kit solutions which the sample could sustain was assigned as the kit number of that specimen. A higher kit number corresponds to high oil resistance and vice versa. To further evaluate the oil resistance, an oil droplet test was performed via a similar method to that used for the water droplet test.

**Characterization and Instruments**

**Thermogravimetric analysis (TGA)**

Thermogravimetric analysis of the reference and obtained materials were performed using a thermogravimetric analyzer (TA Instruments, Q50) by taking a sample with a weight of approximately 7-15 mg and placing it in a nitrogen airflow of 40 mL/min and then heating it from room temperature up to 600 °C at a heating rate of 10 °C/min.

**Differential scanning calorimetry (DSC) analysis**

Differential scanning calorimetry (DSC) tests were performed with a TA Instruments (DSC Q100 model) calorimeter. For these measurements, samples with a mass of ∼10 mg were placed under a nitrogen flow of 100 mL/min. Heating cycles were recorded between the range of 0 and 300 °C at a rate of 10 °C/min.

**
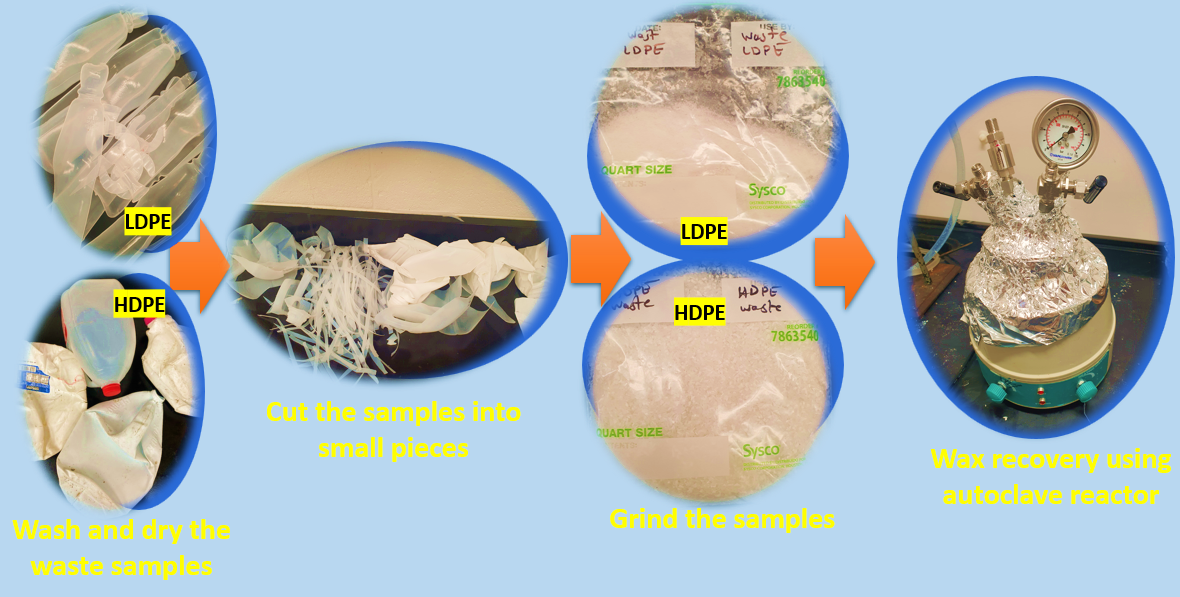
**

**
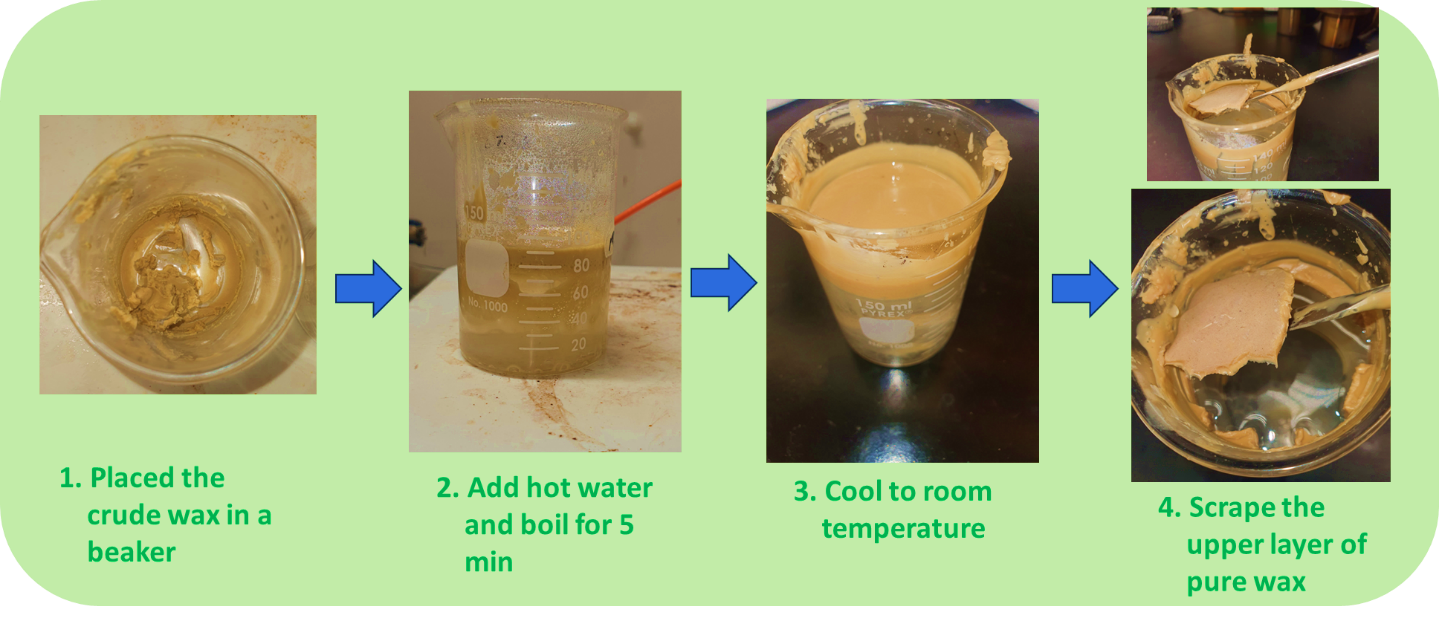
**

**Figure S1** (Top, blue background) Wax formation process performed using waste HDPE/LDPE mixtures as testbed polymers. (Bottom, green background) Stepwise wax recovery and salt separation via purification by hot water treatment.

**Figure S2.** The TGA curves of virgin HDPE/LDPE (50:50) powder (V/50% powder) (black), V/50% powder + Silica (red), and V/50% powder+ salt (blue). It is important to highlight that the rate of byproduct formation accelerates with increasing temperature. Therefore, the reduction in temperature of a HDPE/LDPE blend facilitated by NaCl could be crucial for optimizing wax production.


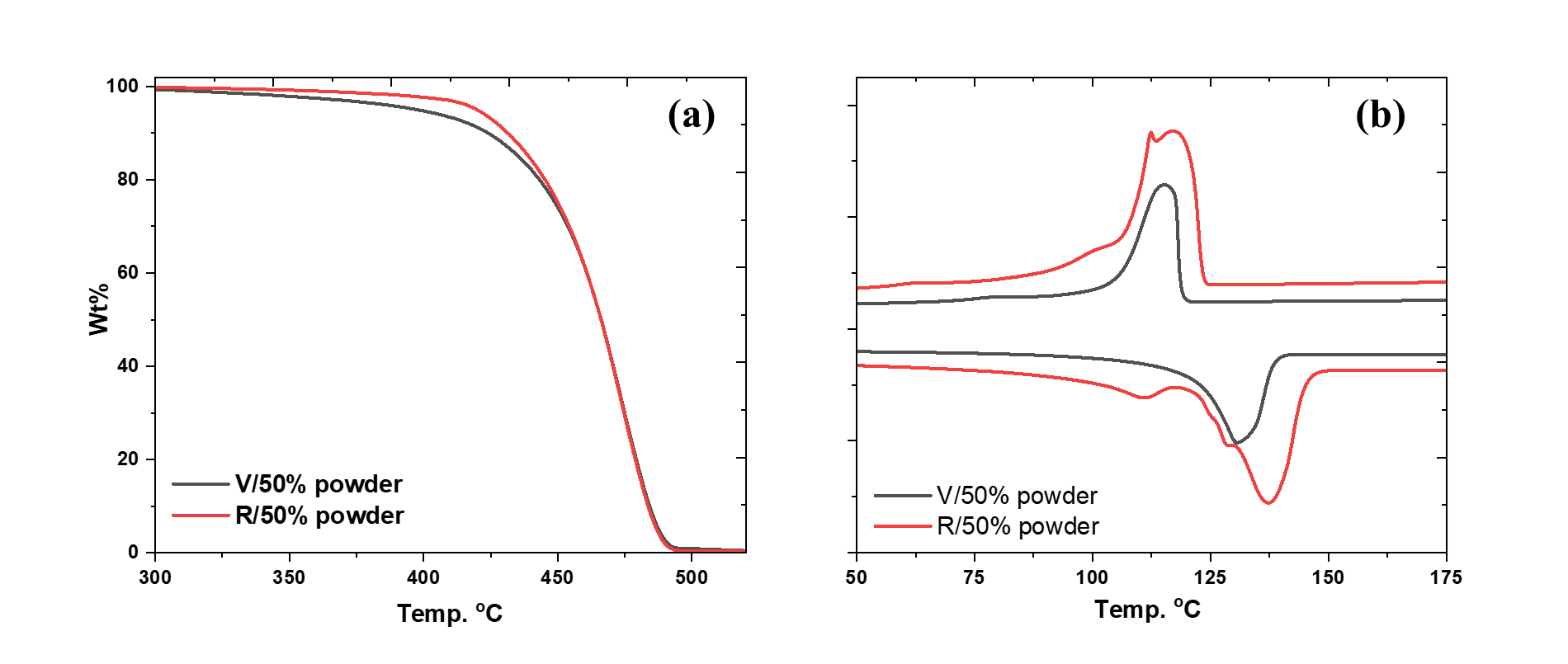


**Figure S3** (a) TGA curves of V/50% and R/50 powders (without pyrolysis) with a heating rate of 10 °C /min under nitrogen atmosphere. (b) DSC curves of V/50% and R/50 powders (without pyrolysis) with a heating rate of 10 °C /min under nitrogen atmosphere.


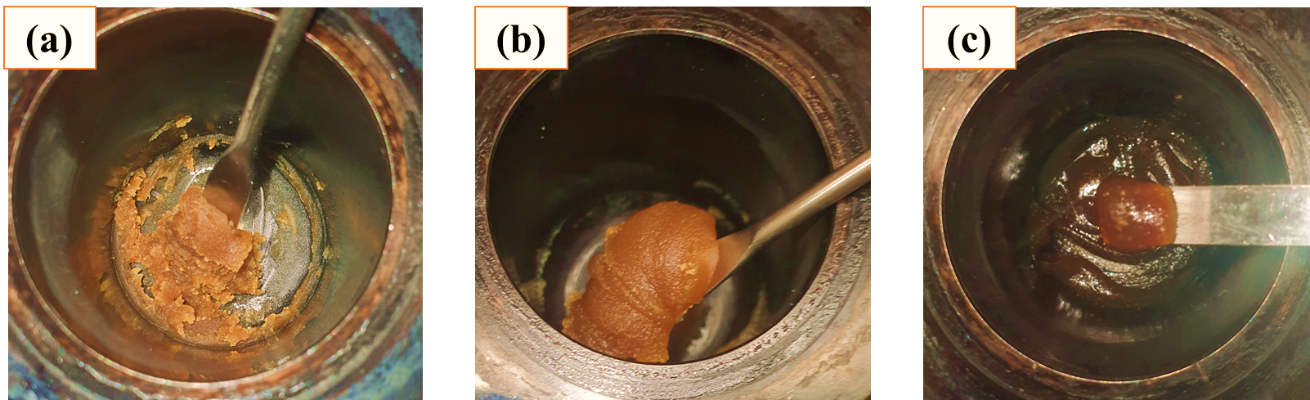


**Figure S4** Photographs of the obtained pyrolytic viscous waxes obtained from virgin HDPE/LDPE mixtures: (a) V3, (b) V4, and (c) V5.


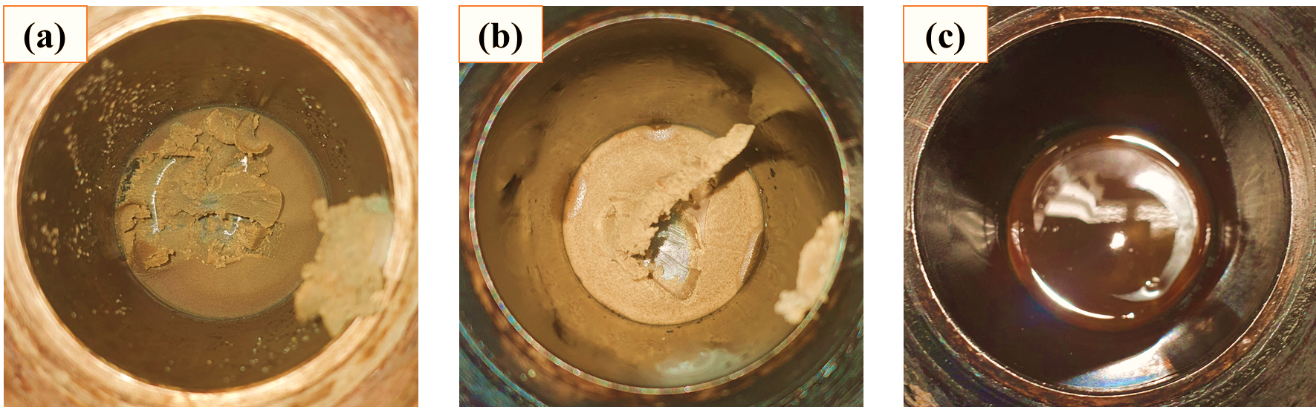


**Figure S5** Photographs of the obtained pyrolytic viscous waxes obtained from waste HDPE/LDPE mixtures: (a) R3, (b) R4, and (c) R5.


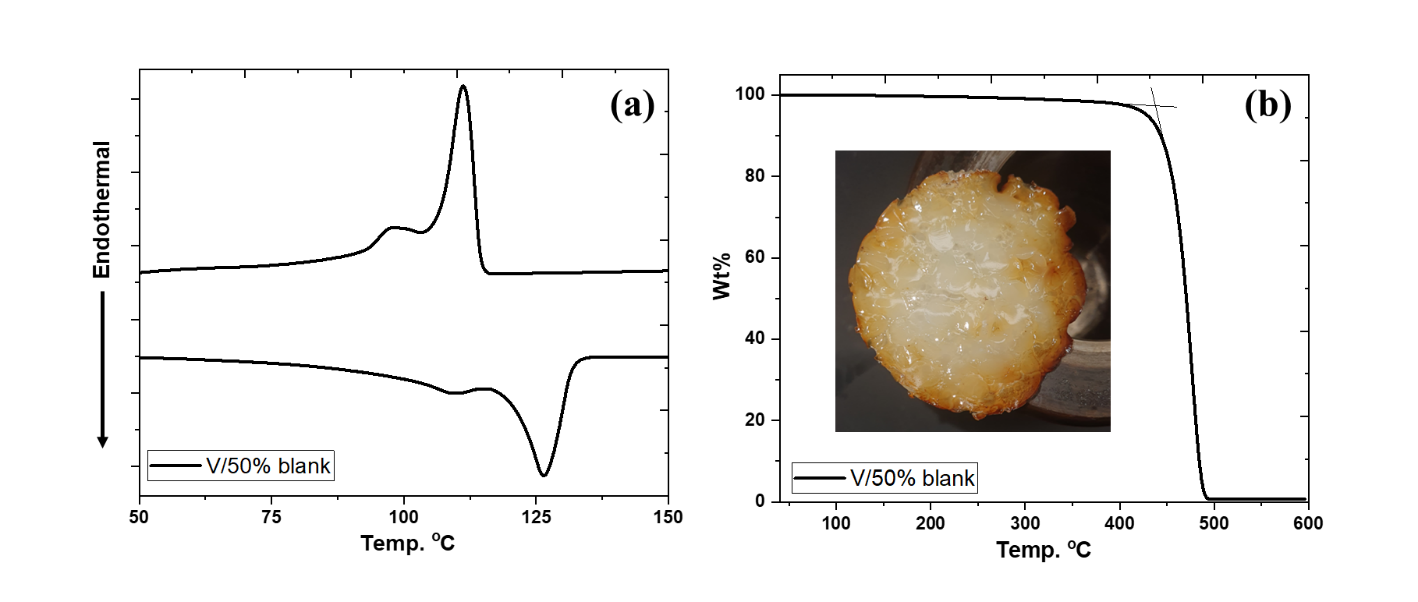


**Figure S6** (a) DSC curve of the pyrolytic solid (V/50% blank) obtained from the HDPE/LDPE (50/50) without NaCl addition; (d) TGA curve of the pyrolytic solid (V/50% blank) obtained from the HDPE/LDPE (50/50) without NaCl addition, with a heating rate of 10 °C /min under nitrogen atmosphere (inset: Photograph of obtained (V/50% blank) solid).


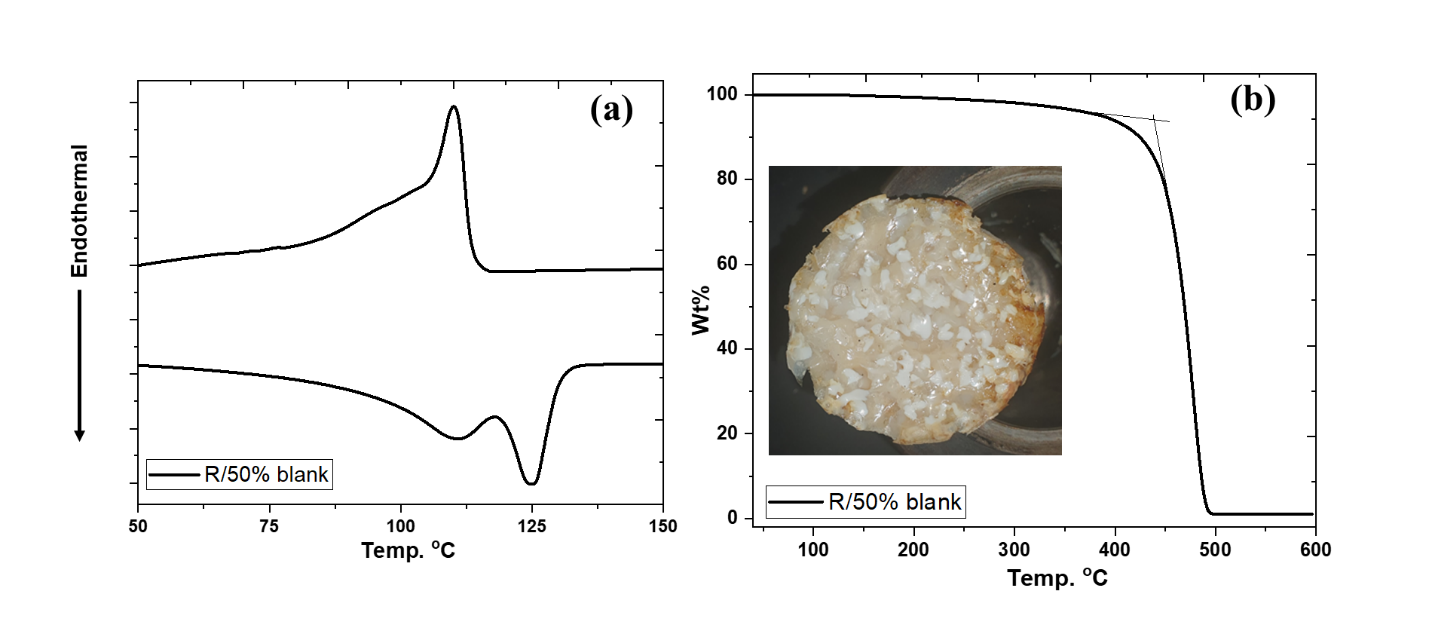


**Figure S7 (**a) DSC curve of the pyrolytic solid (R/50% blank) obtained from the HDPE/LDPE waste (50/50) without NaCl addition; (d) TGA curve of the pyrolytic solid (R/50% blank) from obtained the HDPE/LDPE waste (50/50) without NaCl addition, with a heating rate of 10 °C /min under nitrogen atmosphere (inset: Photograph of obtained (R/50% blank) solid).

**The ^1^H-NMR Spectra**


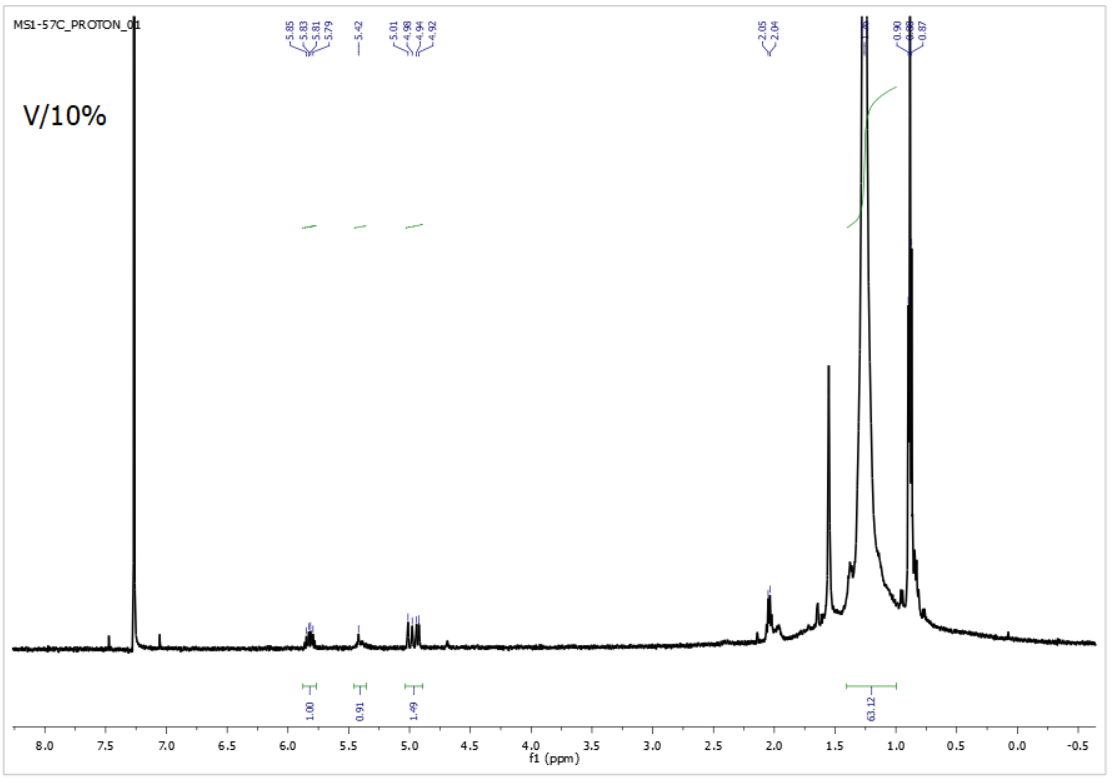


**Figure S8** The ^1^H-NMR spectrum of V/10% wax.


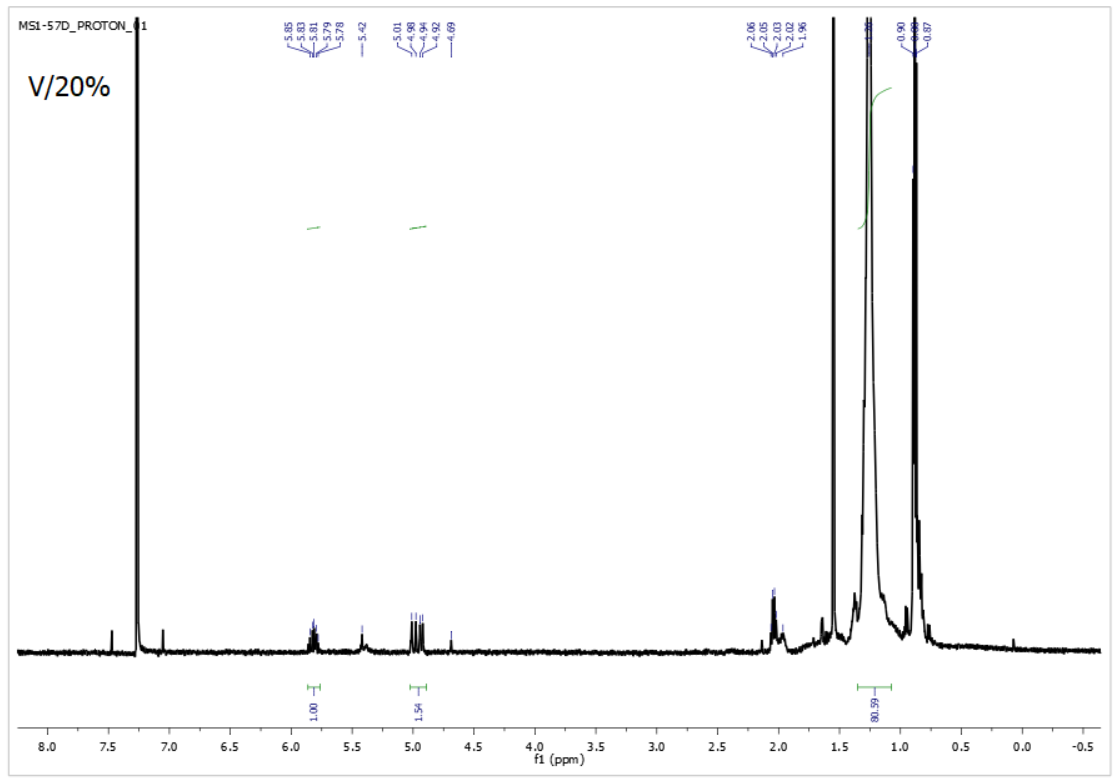


**Figure S9** The ^1^H-NMR spectrum of V/20% wax.


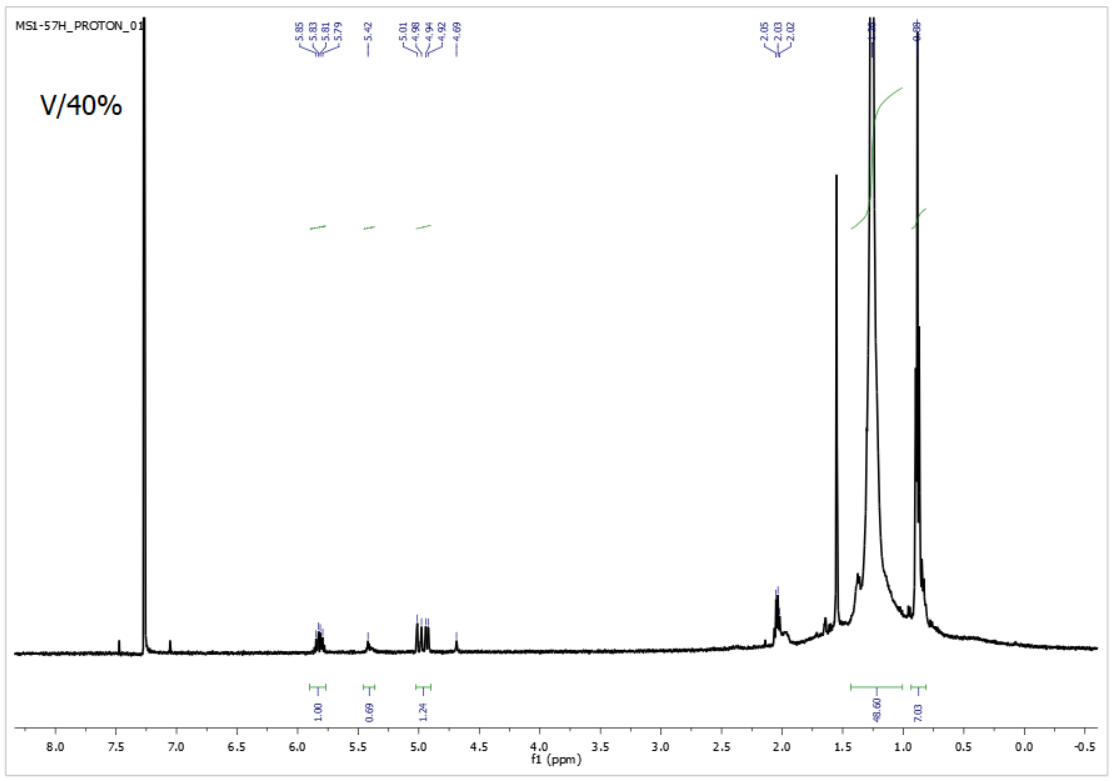


**Figure S10** The ^1^H-NMR spectrum of V/40% wax.


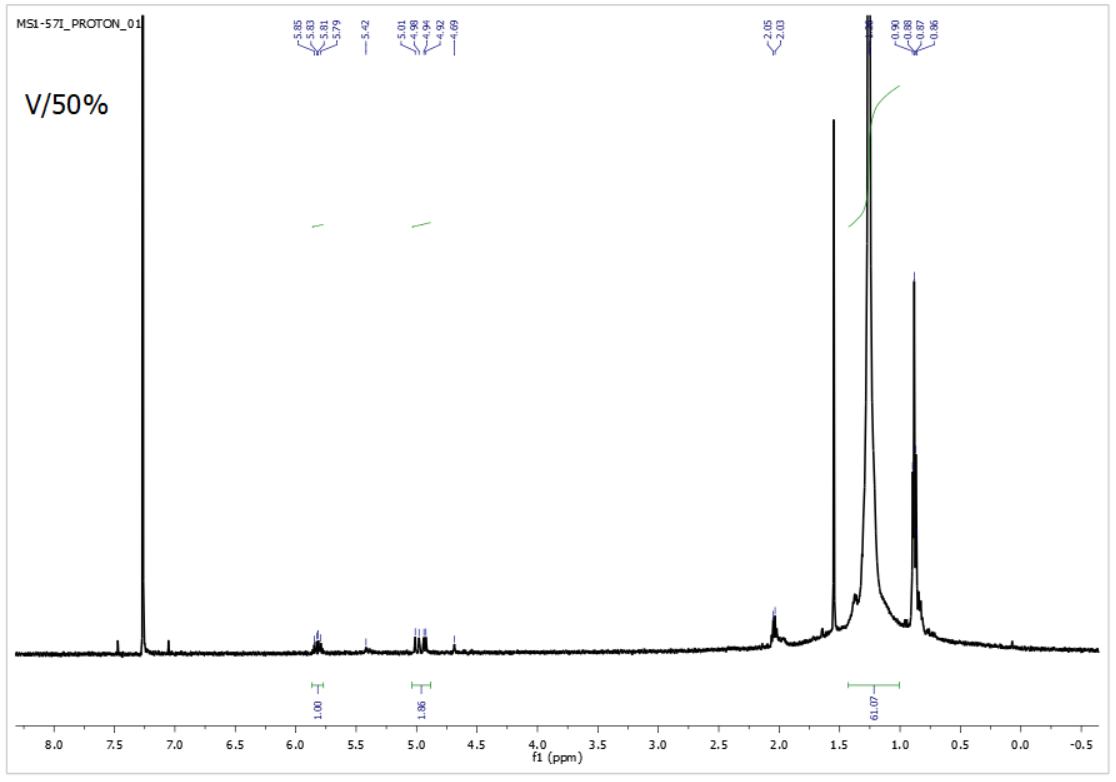


**Figure S11** The ^1^H-NMR spectrum of V/50% wax.


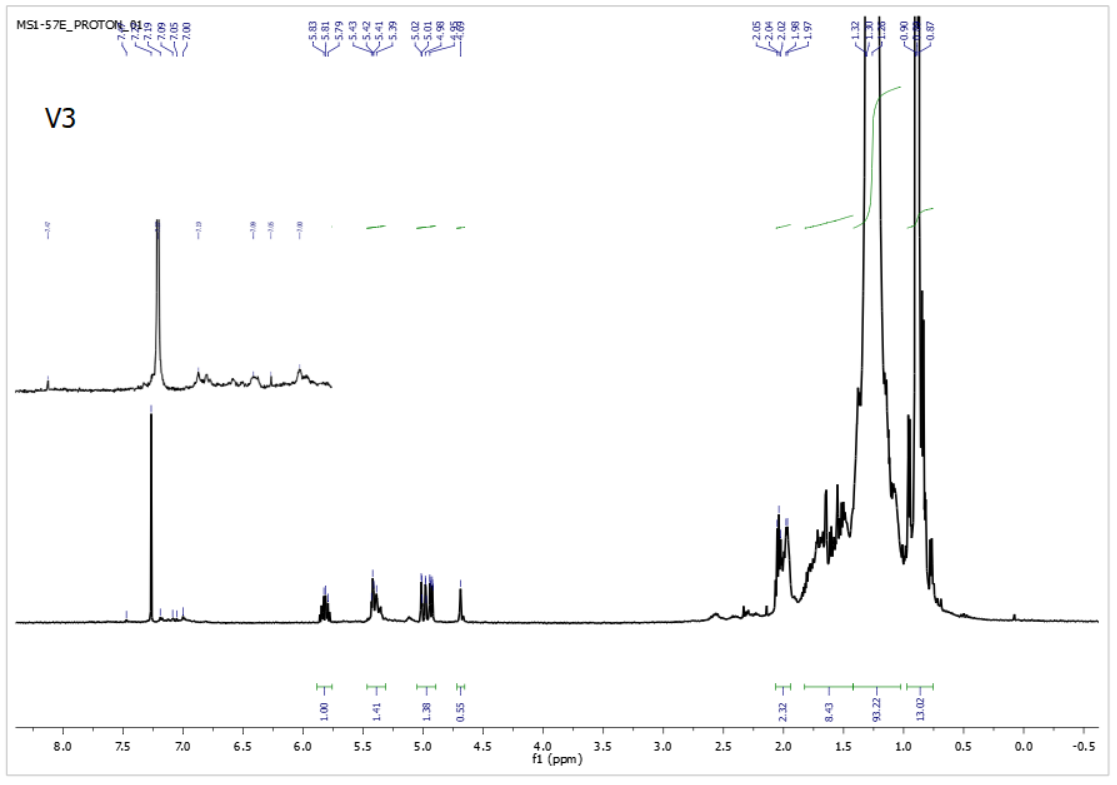


**Figure S12** The ^1^H-NMR spectrum of V3 wax.


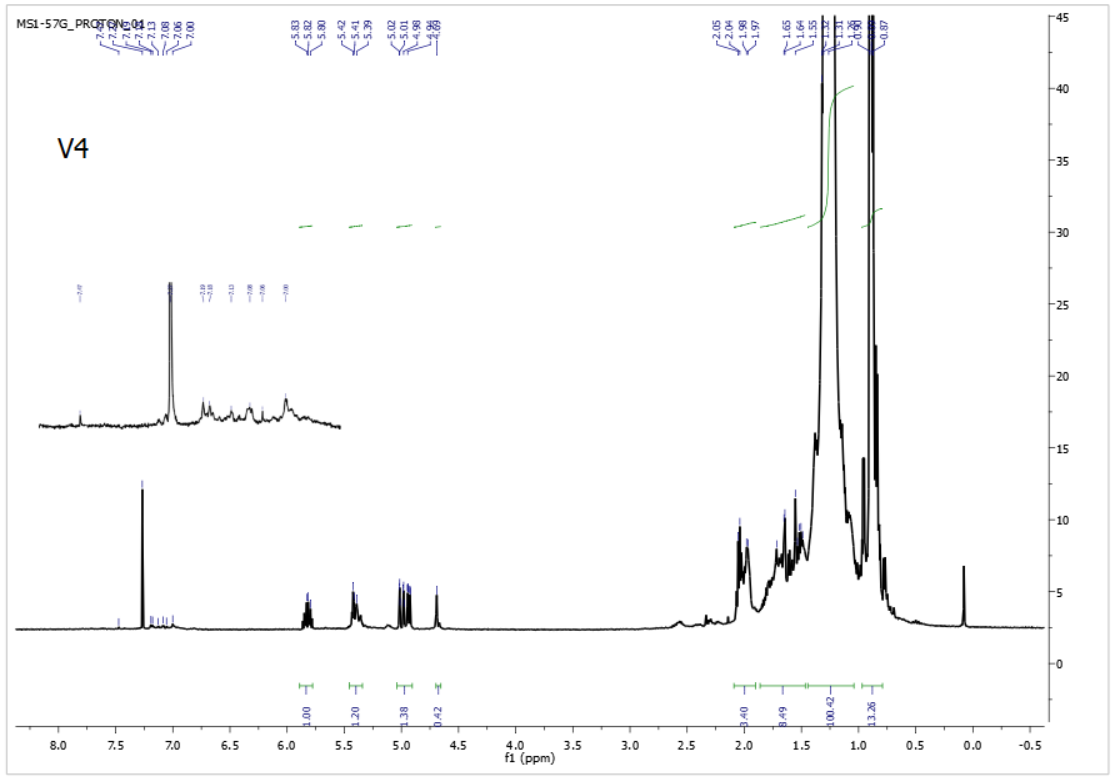


**Figure S13** The ^1^H-NMR spectrum of V4 wax.


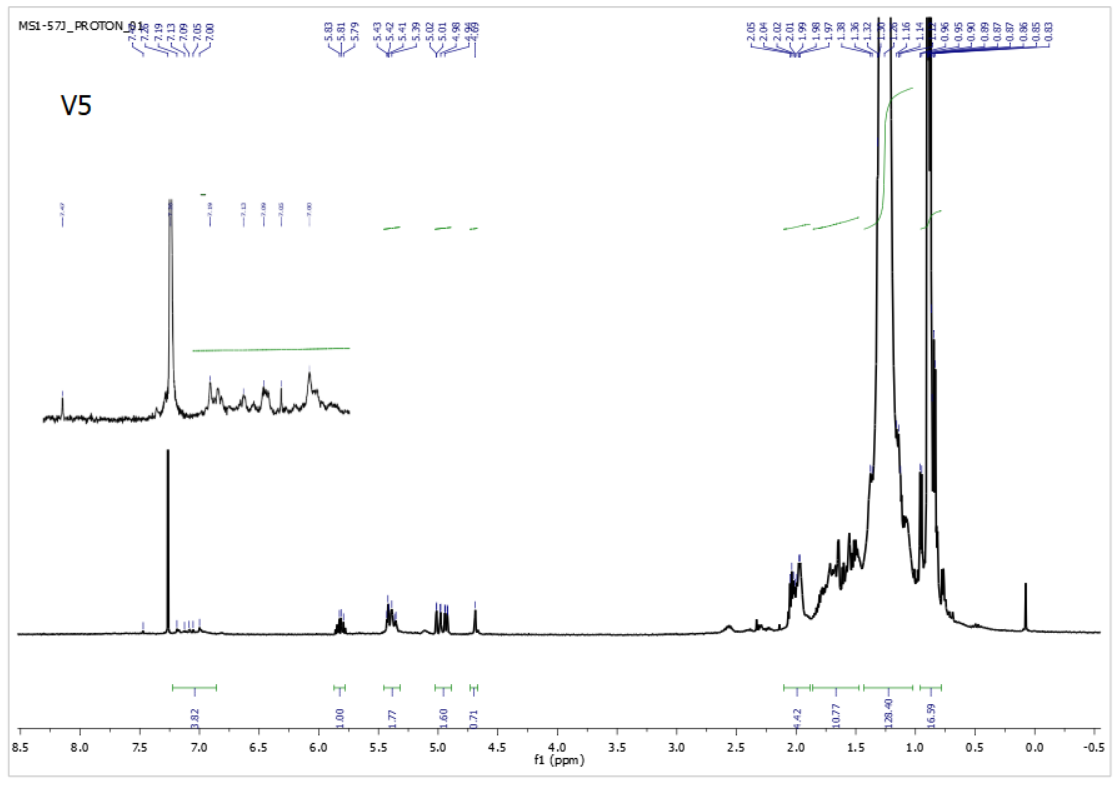


**Figure S14** The ^1^H-NMR spectrum of V5 wax.


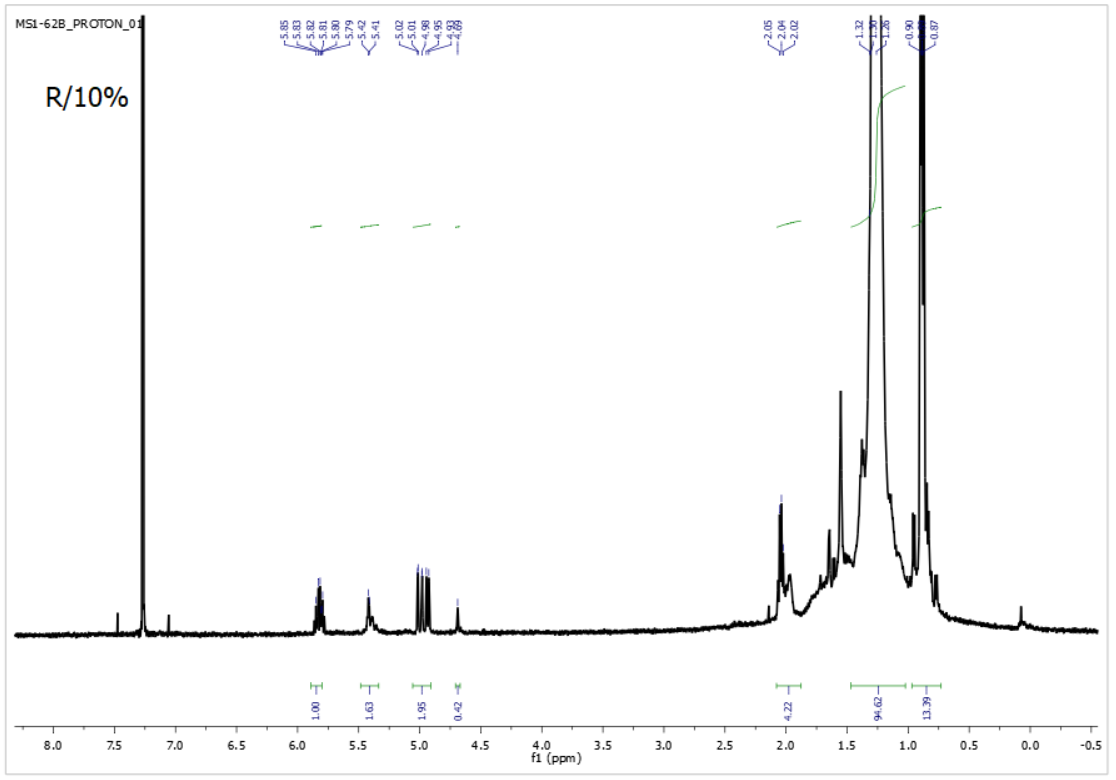


**Figure S15** The ^1^H-NMR spectrum of R/10% wax.


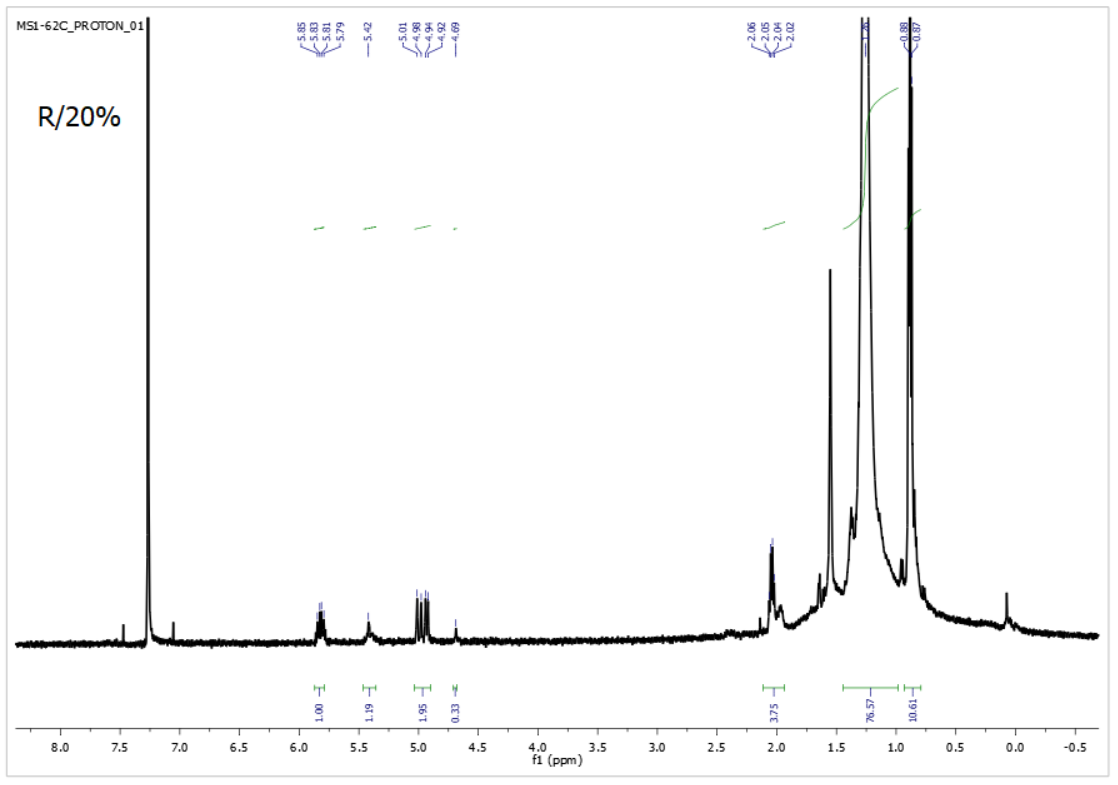


**Figure S16** The ^1^H-NMR spectrum of R/20% wax.


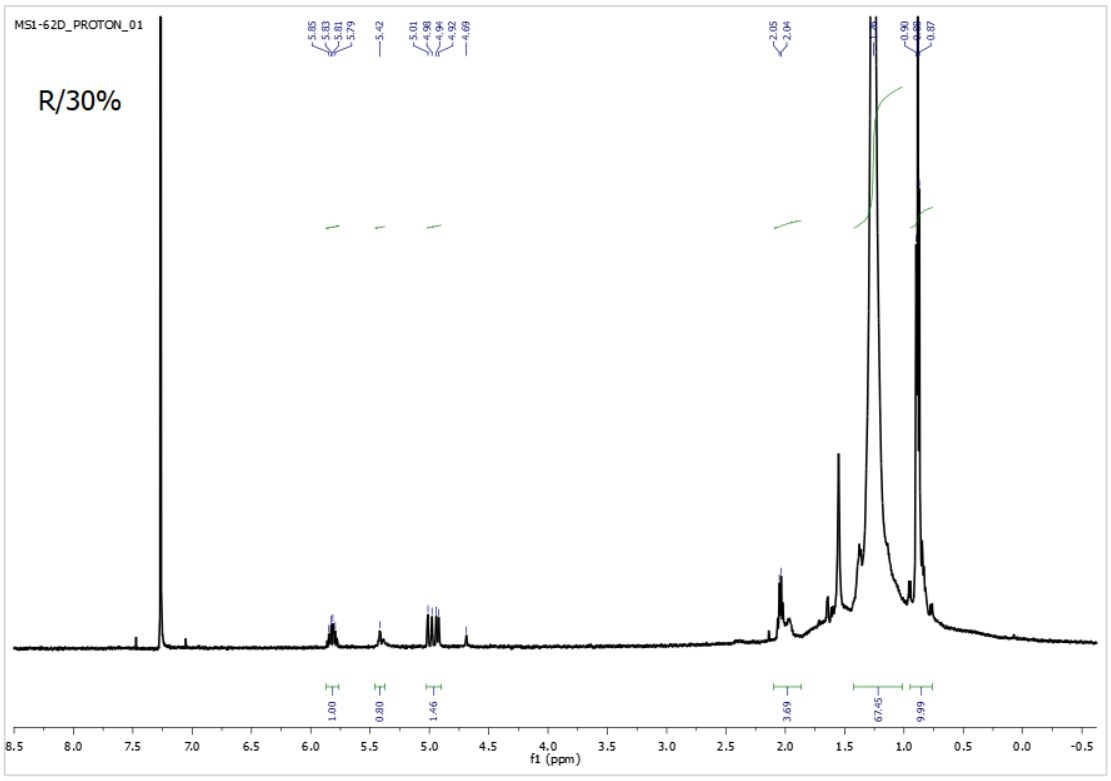


**Figure S17** The ^1^H-NMR spectrum of R/30% wax.


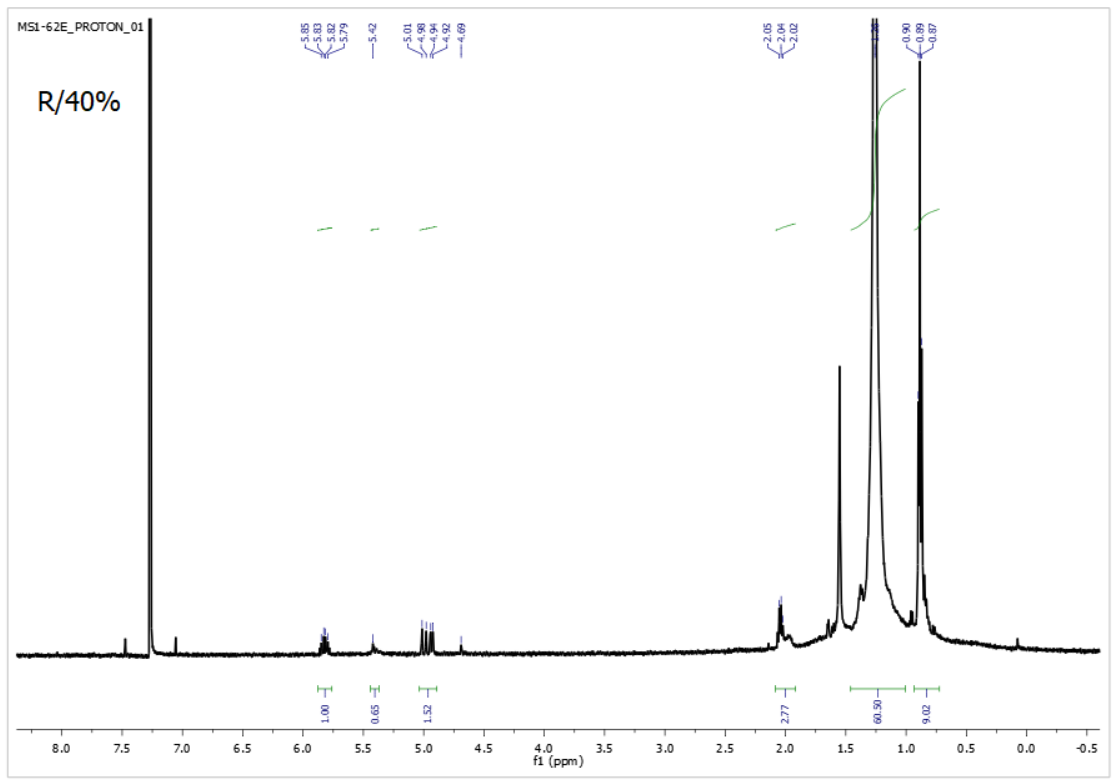


**Figure S18** The ^1^H-NMR spectrum of R/40% wax.


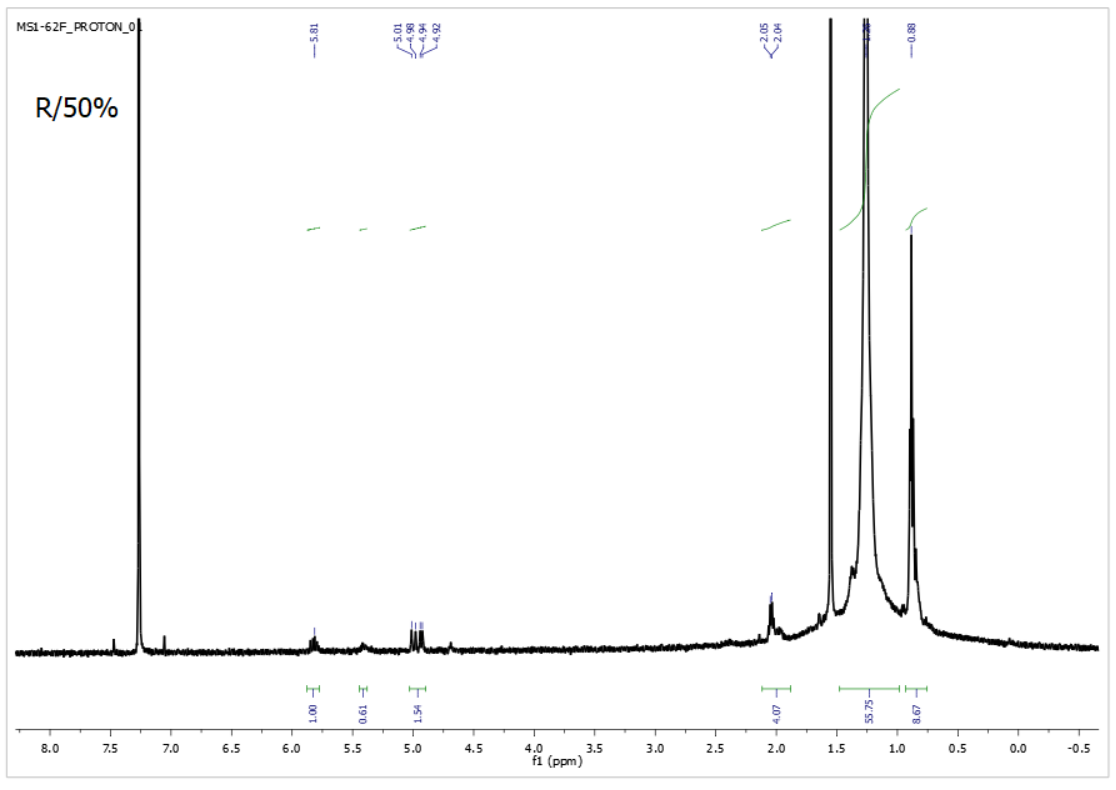


**Figure S19** The ^1^H-NMR spectrum of R/50% wax.


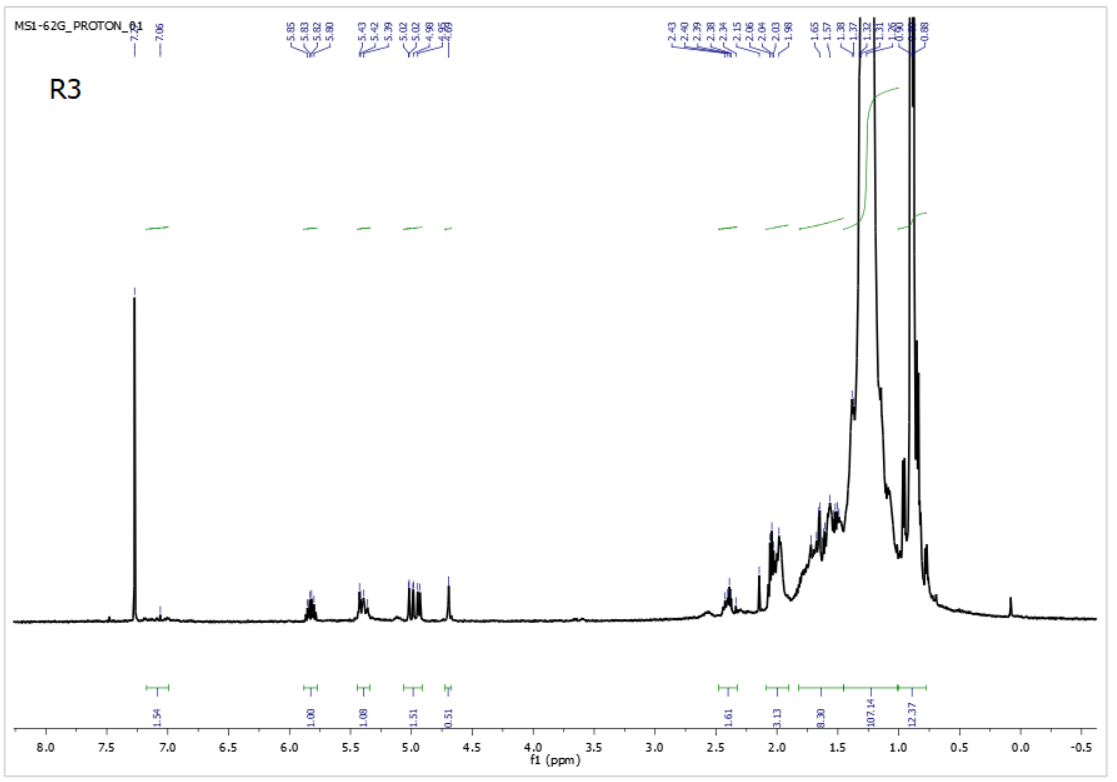


**Figure S20** The ^1^H-NMR spectrum of R3 wax.


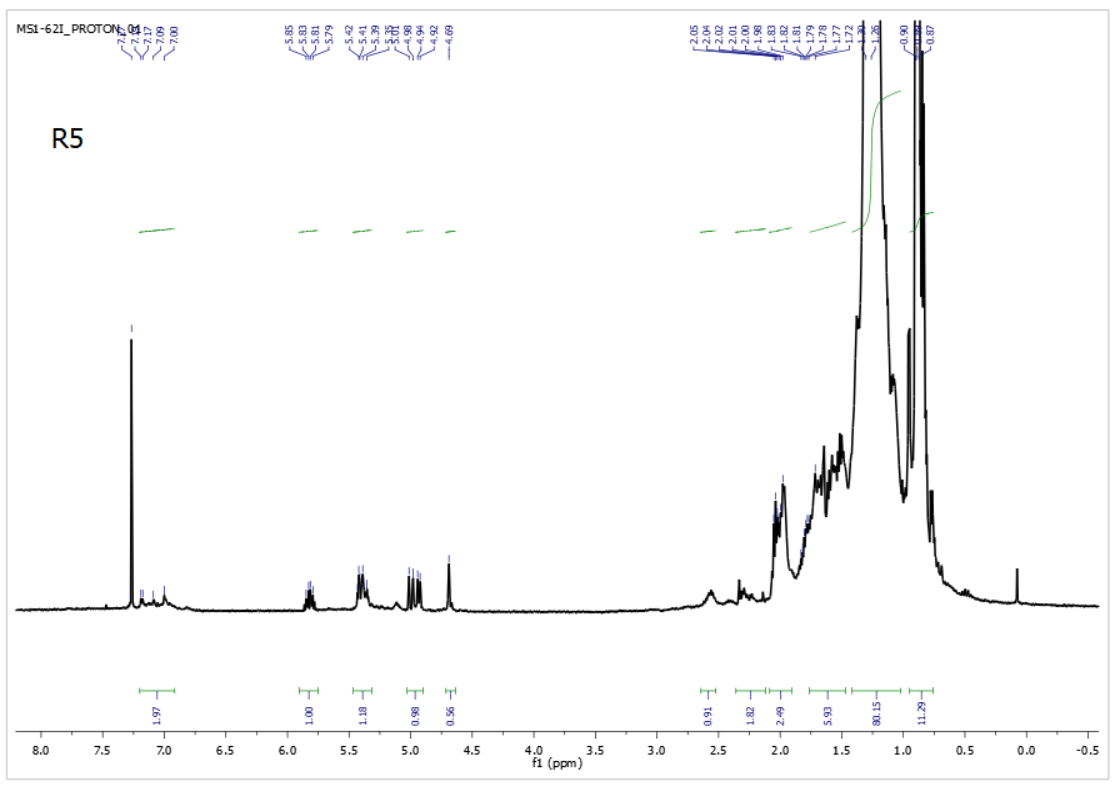


**Figure S21** The ^1^H-NMR spectrum of R5 wax.


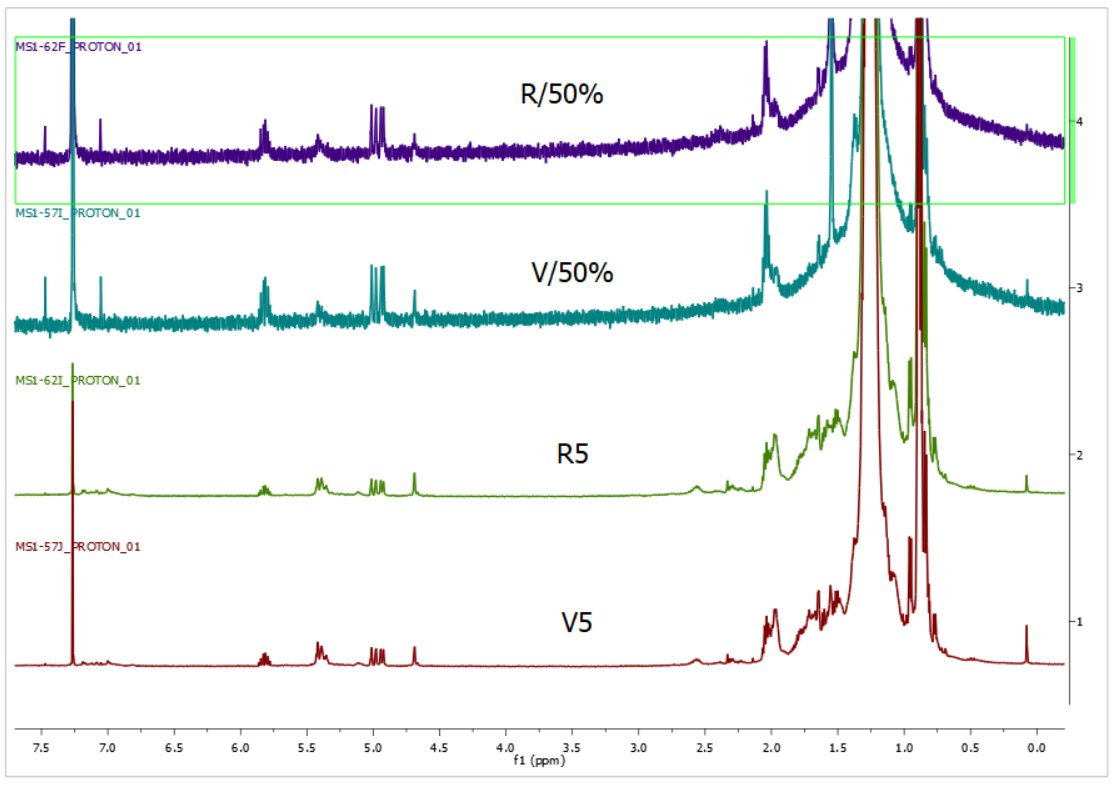


**Figure S22** The ^1^H-NMR spectra of R/50%, V/50%, R5, and V5 waxes.

**The GC-MS Analysis:**


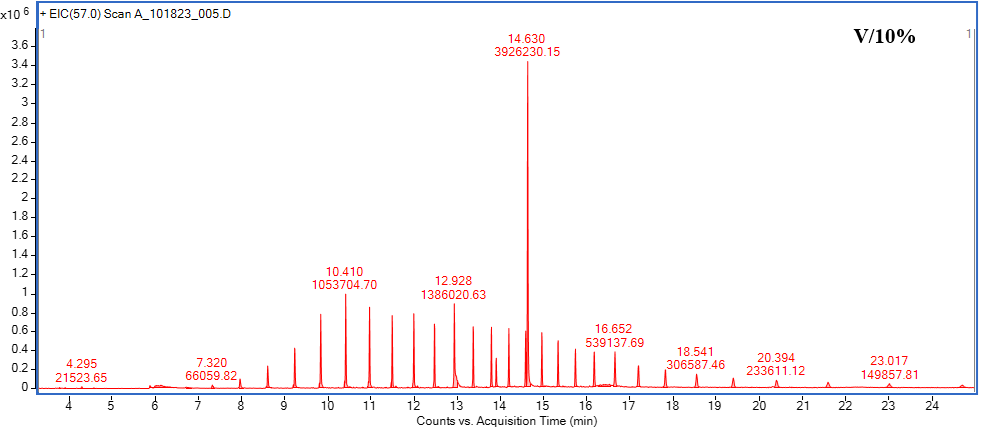


**Figure S23** The GC-MS analysis for the pyrolytic wax V/10%.


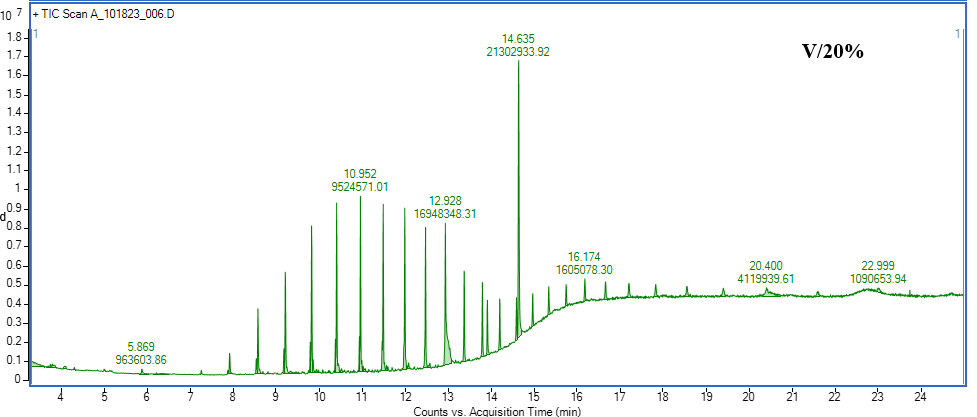


**Figure S24** The GC-MS analysis for the pyrolytic wax V/20%.


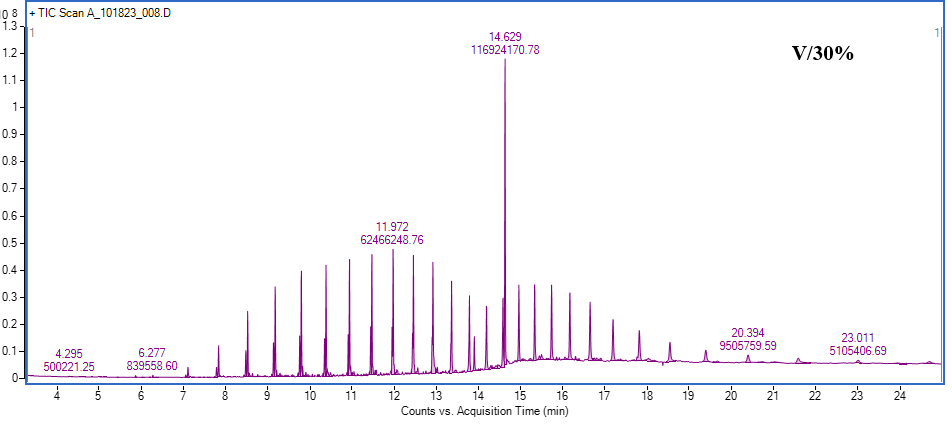


**Figure S25** The GC-MS analysis for the pyrolytic wax V/30%.


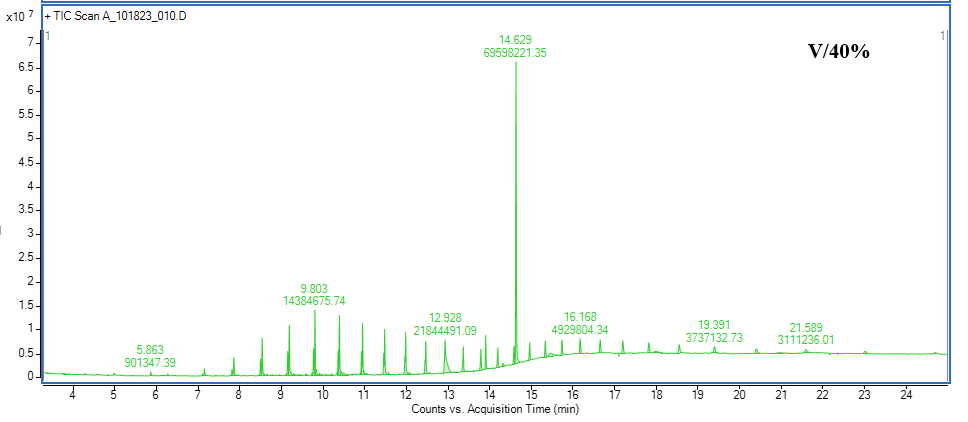


**Figure S26** The GC-MS analysis for the pyrolytic wax V/40%.


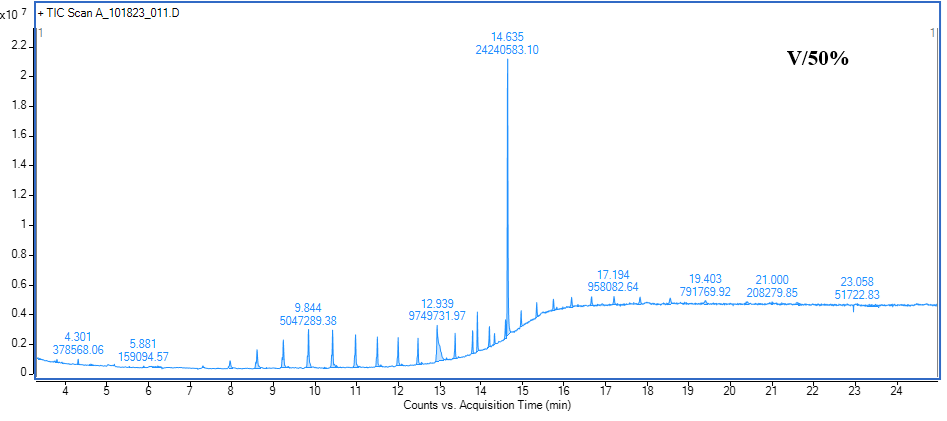


**Figure S27** The GC-MS analysis for the pyrolytic wax V/50%.


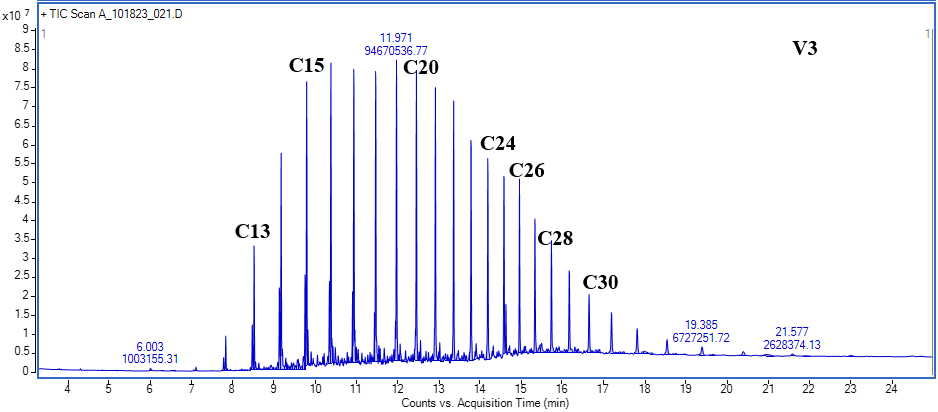


**Figure S28** The GC-MS analysis for the pyrolytic viscous wax V3.


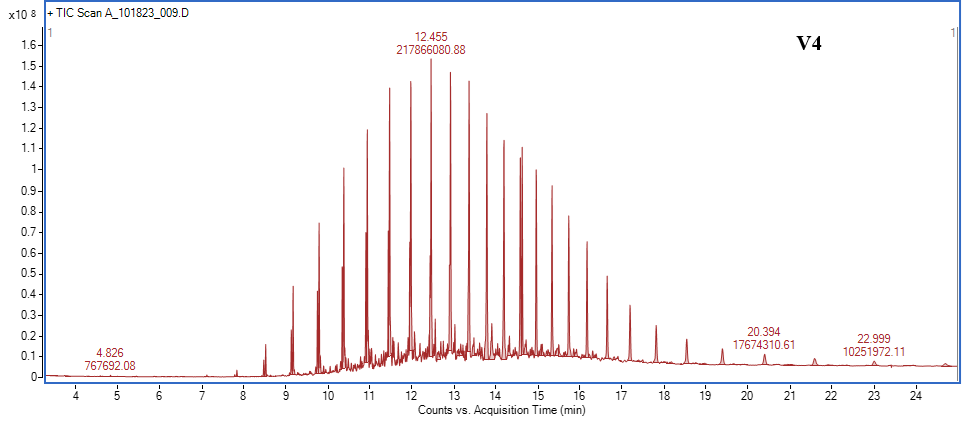


**Figure S29** The GC-MS analysis for the pyrolytic viscous wax V4.


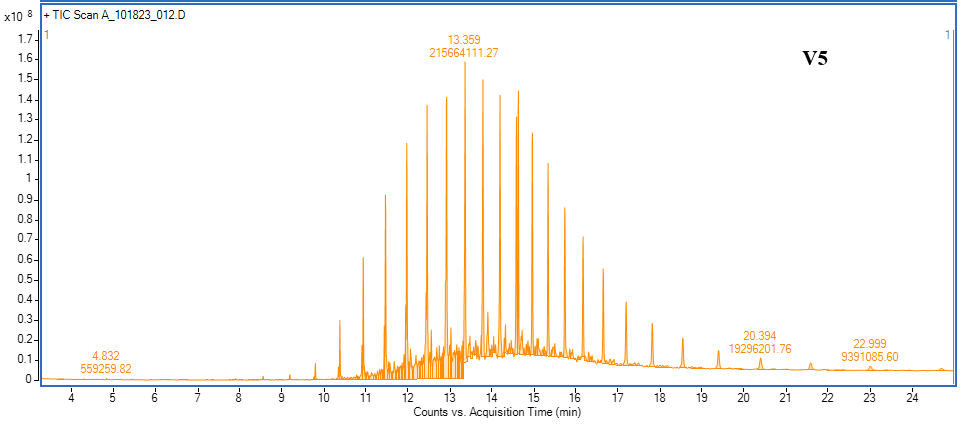


**Figure S30** The GC-MS analysis for the pyrolytic viscous wax V5.


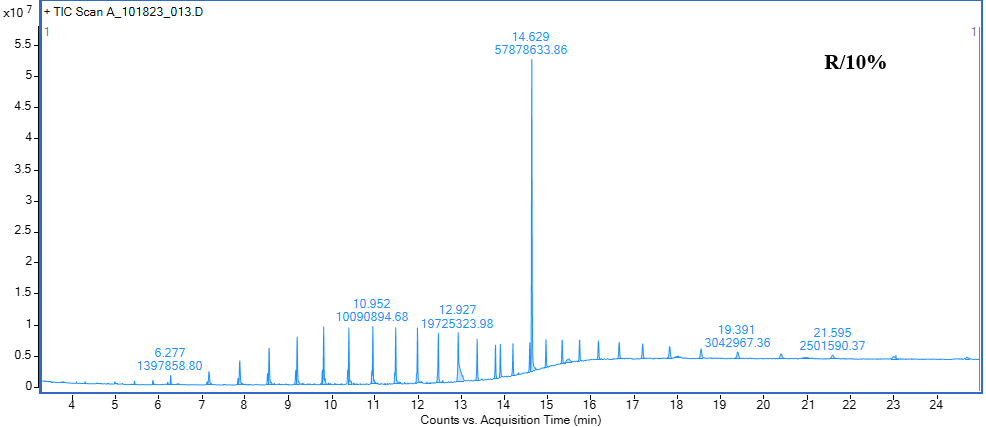


**Figure S31** The GC-MS analysis for the pyrolytic wax R/10%.


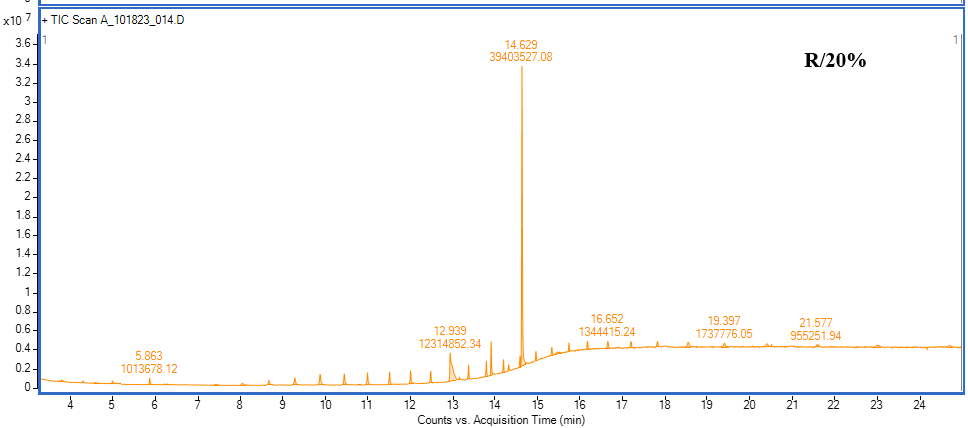


**Figure S32** The GC-MS analysis for the pyrolytic wax R/20%.


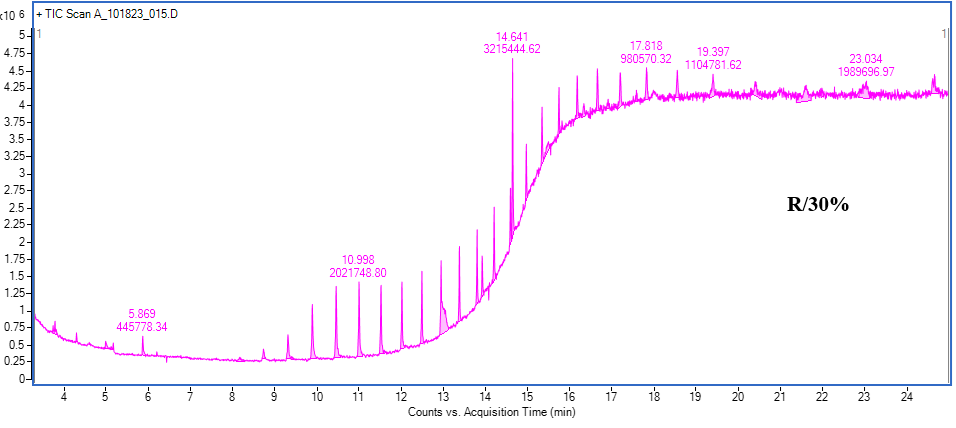


**Figure S33** The GC-MS analysis for the pyrolytic wax R/30%.


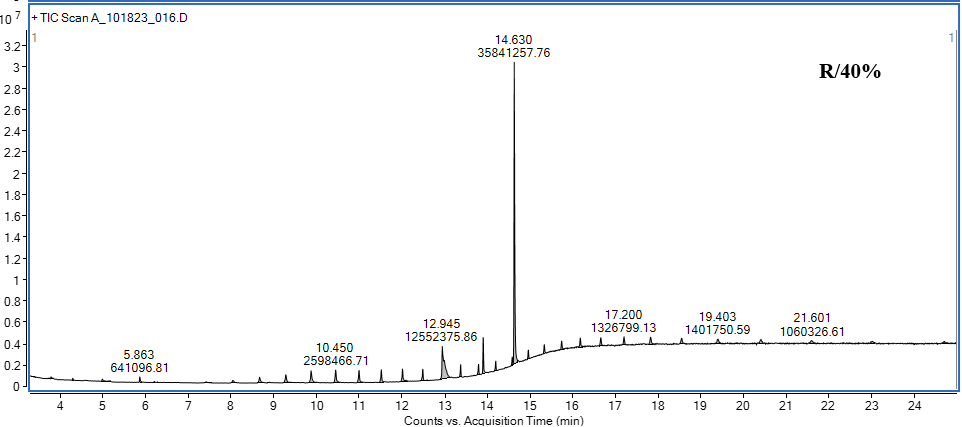


**Figure S34** The GC-MS analysis for the pyrolytic wax R/40%.


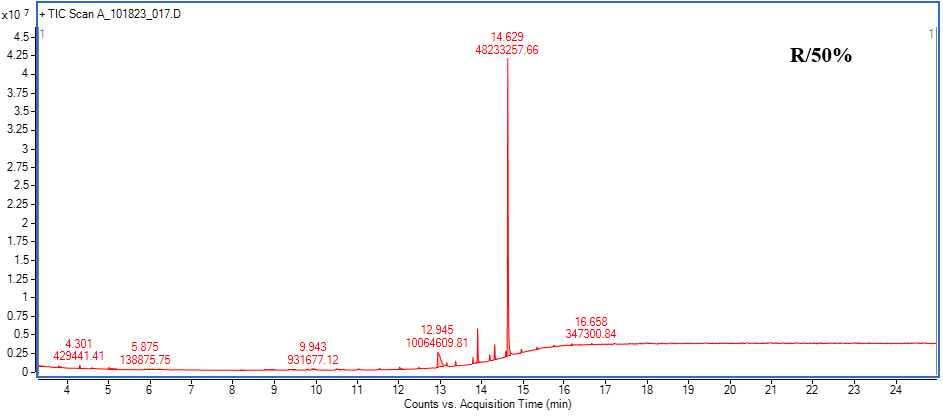


**Figure S35** The GC-MS analysis for the pyrolytic wax R/50%.


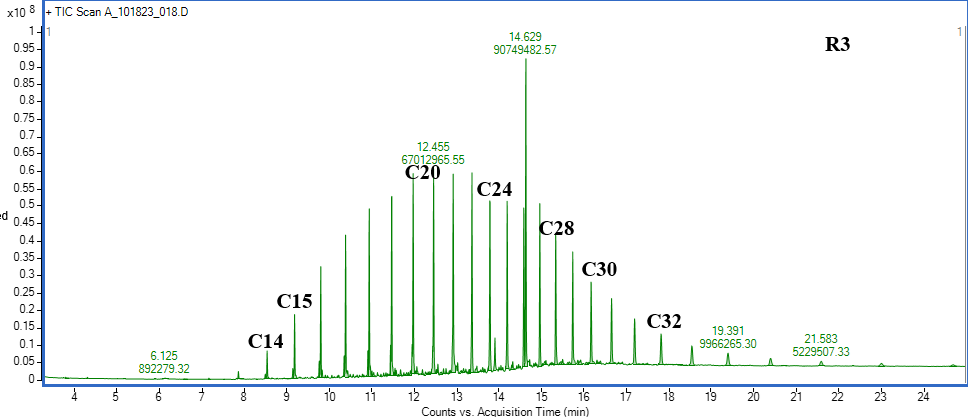


**Figure S36** The GC-MS analysis for the pyrolytic viscous wax R3.


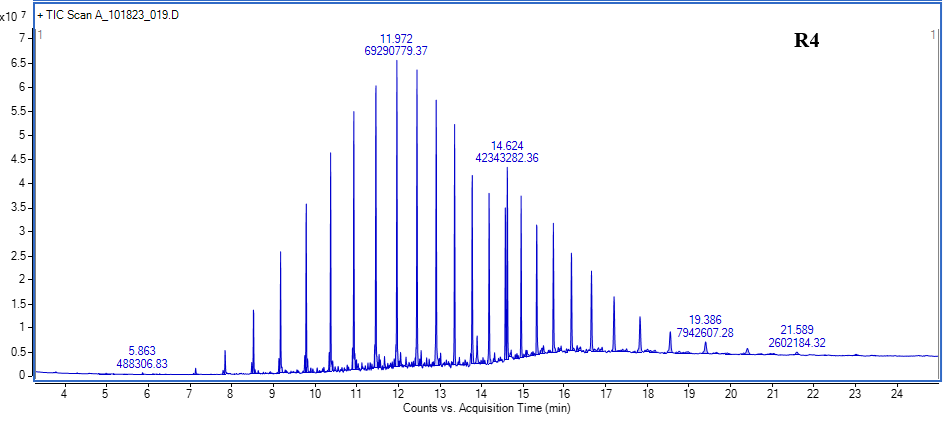


**Figure S37** The GC-MS analysis for the pyrolytic viscous wax R4.


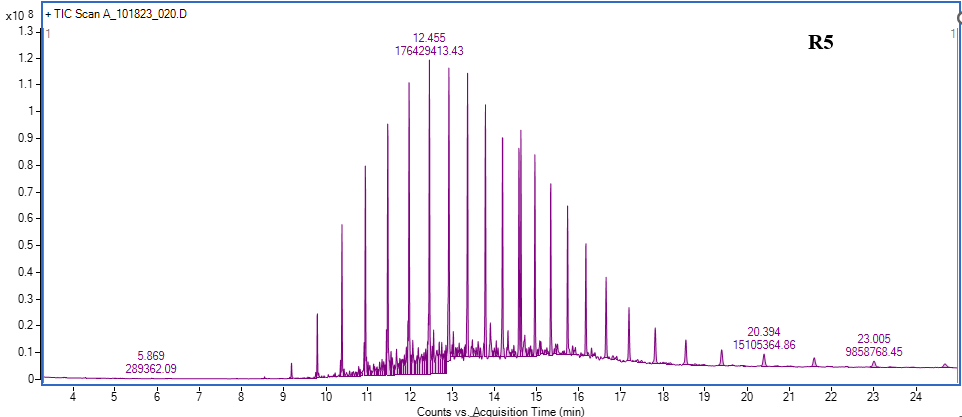


Figure S38 The GC-MS analysis for the pyrolytic viscous wax R5.


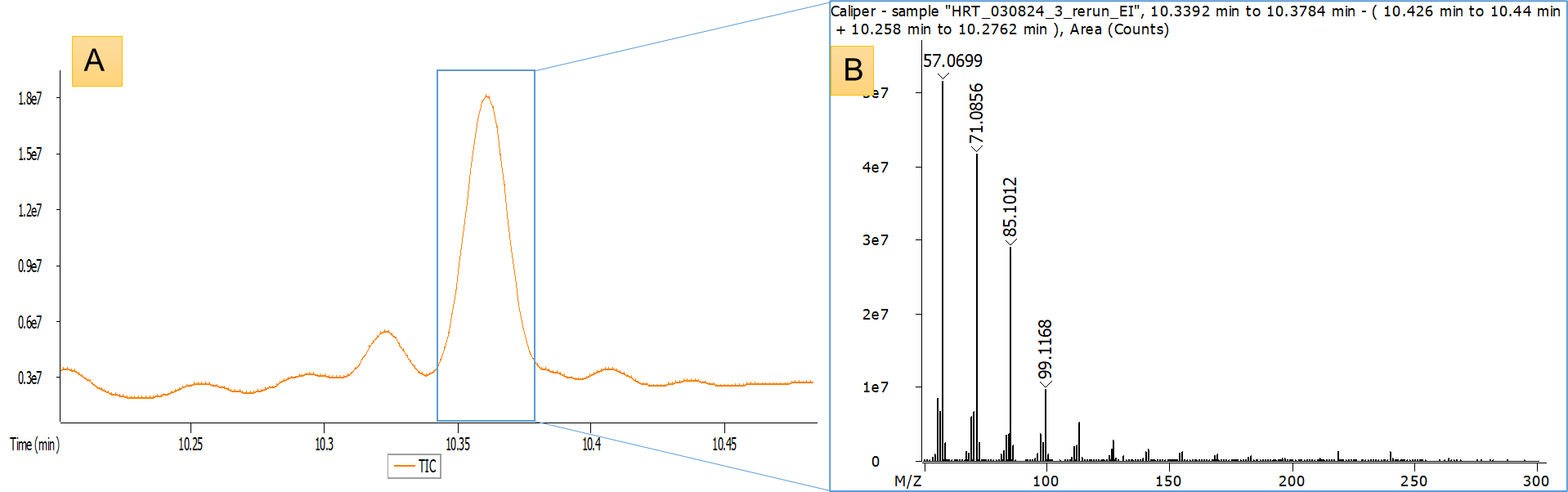


Figure S39. A) The GC-MS analysis for the pyrolytic viscous wax R3, Extracted ion chromatogram of C17; B. MS fragmentation patterns of peak 10.36 min.


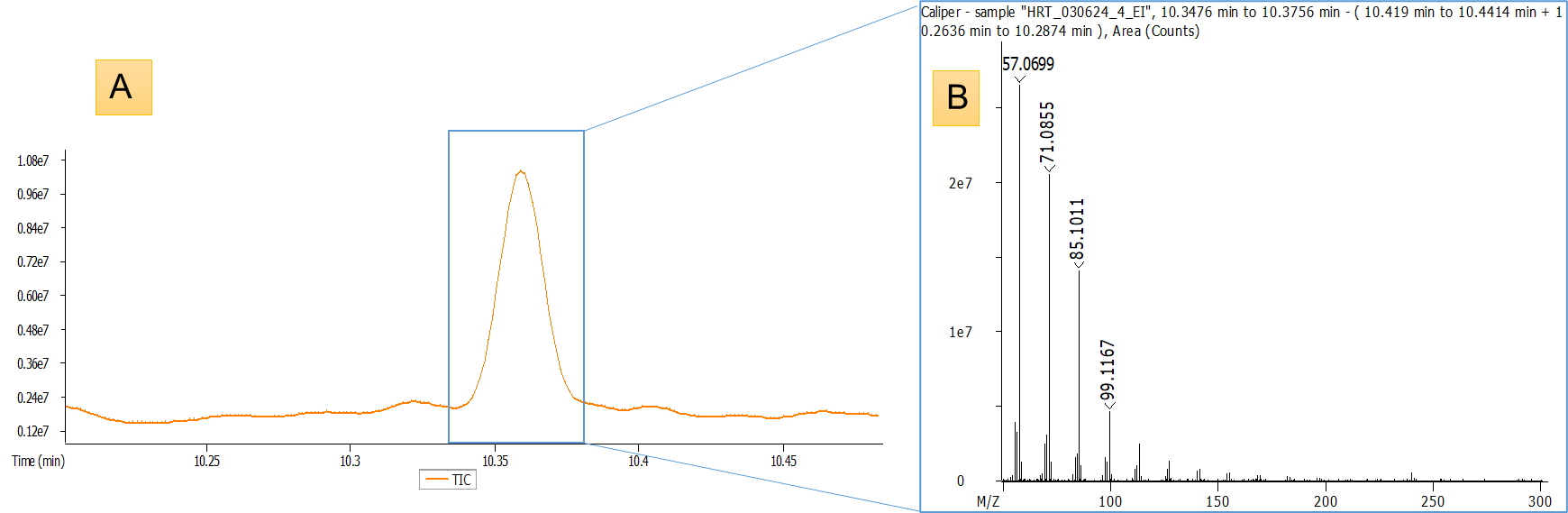


Figure S40. A) The GC-MS analysis for the pyrolytic viscous wax R3 (without the use of NaCl), Extracted ion chromatogram of C17; B. MS fragmentation patterns of peak 10.36 min.

Table S1.

The parameters for the economic analysis for the system with 90% and 95% wax recovery.

peak 10.36 min

|  | **Revenue at 90% wax** | **Revenue at 95% wax** | **Added costs** |
| --- | --- | --- | --- |
|  | $/y |  | $/y |
| Oil | 0 | 0 |  |
| Gas | 105,181 | 52,590 |  |
| Wax | 15,120,000 | 15,951,600 |  |
| Salt |  |  | 212,966 |
| Plastic |  |  | 184,800 |
| **Net Revenue** | **14,827,415** | **15,606,424** | **397,766** |

**Table S2.**

Inputs used to create our revenue model.

| **Inputs** | **Units** | **Base** |
| --- | --- | --- |
| Feed rate | *tonnes/h* | 1 |
| Reaction time | *h* | 4 |
| Wax yield | *wt%* | 90-95 |
| Waste rigid HDPE (cost) | *$/lb* | 0.01 |
| Gas yield | *wt%* | 5-10 |
| NaCl added | *wt% of feed* | 10 |
| NG LHV | *MJ/kg* | 41 |
| PE specific heat | *kJ/kg-C* | 2 |
| Reactor temperature | *C* | 425 |
| Flue gas temperature | *C* | 450 |
| Paraffin specific heat | *kJ/kg-C* | 2.5 |
| Paraffin heating value | *MJ/kg* | 42 |
| Gas heating value | *MJ/kg* | 50 |
| Selling price gasoline | *$/gal* | 3.65 |
| Selling price natural gas | *$/MMBtu* | 2.64 |
| Selling price NaCl | *$/ton* | 230 |
| Selling price Wax | *$/kg* | 2 |
| Operating days/year | *days/y* | 350 |
| Density gasoline | *kg/L* | 0.72 |

**Table S3.**

Revenue estimates for different wax and gas fractions obtained.

|  | **Revenue** | **Added costs** |
| --- | --- | --- |
|  | $/y | $/y |
| **This work at 90% wax production** | | |
| Oil | 0 |  |
| Gas (10) % | 105,181 |  |
| Wax (90%) | 15,120,000 | Mixed Plastic Waste cost= 184,800 |
| Salt |  | 212,966 |
| Net Revenue | **14,827,415** |  |
|  | | |
| **This work at 95% wax production** | | |
|  | **Revenue** | **Added costs** |
|  | $/y | $/y |
| Oil | 0 |  |
| Gas (5)% | **52,590** |  |
| Wax (95%) | **15,951,600** |  |
| Salt |  | 212,966 |
| Mixed plastics cost |  | Mixed Plastic Waste cost =184,800 |
| Net Revenue | **15,606,424** |  |
|  |  |  |
|  | | |
| **State of the art (literature) at 32% wax production^1^** | | |
|  | **Revenue** | **Added costs** |
|  | $/y | $/y |
| Oil | 0 |  |
| Gas (62)% | **683,675** | 212,966 |
| Wax (32%) | **5,376,000** |  |
| Salt |  | 0 |
| Plastic |  | HDPE Plastic Waste cost= 924,000 |
| Net Revenue | **4,922,709** |  |

**References**

1. S. Al-Salem, A. Dutta, Wax Recovery from the Pyrolysis of Virgin and Waste Plastics. *Ind. Eng. Chem. Res.* **60**, 8301-8309 (2021). doi: 10.1021/acs.iecr.1c01176
